# Rab11A Depletion in Microglia-Derived Extracellular Vesicle Proteome upon Beta-Amyloid Treatment

**DOI:** 10.1007/s12013-023-01133-4

**Published:** 2023-03-30

**Authors:** Giuseppina Mignogna, Cinzia Fabrizi, Virginia Correani, Alessandra Giorgi, Bruno Maras

**Affiliations:** 1 Dipartimento di Scienze Biochimiche, Sapienza Università di Roma, Rome, Italy; 2 Dipartimento di Scienze, Anatomiche Istologiche Medico-Legali e dell’Apparato Locomotore Sapienza Università di Roma, Rome, Italy

**Keywords:** Microglia, Proteomics, Extracellular vesicles, Alzheimer’s disease, Rab11A

## Abstract

Microglia, the macrophage-like glial cells, behave as sentinels against exogenous pathogens invading the neural tissue. Their commitment is not only confined to the defensive function, but they also perform balancing trophic activities such as neuronal postnatal development, remodeling and pruning of synapses. Likewise, microglia-derived extracellular vesicles (EVs) can play strategic roles in maintaining a healthy brain by modulating neuronal activity and by controlling neurite outgrowth as well as innate immune response. Nevertheless, strong evidence also points to their role in the development of neurodegenerative pathologies such as Alzheimer’s disease (AD). Here, we explored EV protein content released by BV2 microglial cells in a resting state and after stimulation with beta-amyloid peptides (Aβ), mimicking conditions occurring in AD. In the resting BV2 cells, we extended the list of proteins present in mouse microglia EV cargo with respect to those reported in the Vesiclepedia exosome database while, in amyloid-triggered microglia, we highlighted a pronounced drop in EV protein content. Focusing on Rab11A, a key factor in the recycling routes of amyloid species, we observed a dramatic decrease of this protein in Aβ-treated microglia EV cargo with respect to the EVs from the untreated sample. This decrease might affect the delivery of Rab11A to neurons thus increasing the harmful amyloid burden in neuronal cells that eventually may lead to their death. We tentatively proposed that alterations observed in EVs derived from Aβ-treated microglia may represent molecular features that, among others, shape the disease-associated microglial phenotype, a recently proposed subset of microglial population, present in neurodegenerative pathologies.

## Introduction

Cell-to-cell communication is a major issue for the comprehension of the dynamic events occurring among cells. Extracellular vesicles (EVs) represent versatile carriers for molecular cargo due to their minute structure that enable these particles to move easily from the site of release to cells localized nearby [1]. EVs can also influence the metabolic fate of recipient cells localized far from their place of origin by diffusion in biological fluids. The term EVs includes three different vesicle types: apoptotic bodies, microvesicles (MVs), and exosomes. These particles differ in origin and size with apoptotic bodies (800–5000 nm) released after a cellular apoptotic event, MVs (100–1000 nm) originating from plasma membrane budding, and exosomes (40–150 nm) assembled in endosomal compartment [2]. In the Central Nervous System (CNS), EVs produced by glial cells [3, 4] and neurons [5] are a way to maintain crosstalk among these resident cells, preserve the correct homeostasis of the tissue, and quickly respond to local environmental alterations.

Microglia, the macrophage-like glial cells, behave as sentinels against exogenous pathogens invading the neural tissue [6]. Their commitment is not only confined to the defensive function, but they also perform trophic activities by assisting neuronal postnatal development [7] and by refining synapses through remodeling and pruning [8]. Likewise, microglia-derived EVs can enact a strategic role in modulating neuronal activity and in controlling neurite outgrowth as well as in priming innate immune response. The former activities seem to reside on EV plasma membrane surface components able to induce a variation of miniature excitatory postsynaptic current on neuronal recipient cells. They also enhance the synthesis of ceramide and sphingosine that in turn upregulate synaptic activity [9] and through thrombospondin-1 and thrombospondin-4 may trigger neurite outgrowth and synaptogenesis [10]. In addition, EVs can provide a metabolic contribution to neurons considering their enrichment in glycolytic enzymes such as lactate dehydrogenase, pyruvate kinase and, glyceraldehyde 3-phosphate dehydrogenase [10]. As far as innate immune response is concerned, EVs are considered important components in inflammatory and neuroimmune interactions as well as coordinators of the immune response [11, 12]. Nevertheless, although a relevant role in maintaining health has been demonstrated for EVs, strong evidence points also to their role in the development of neurological diseases [13, 14]. EVs may exacerbate neurodegenerative pathologies such as Alzheimer’s disease (AD) with the propagation of toxic beta-amyloid peptides (Aβ) [15, 16] and Tau aggregates [17] and in triggering and maintaining a chronic inflammatory environment [18]. In AD, Aβ can build supramolecular structures that lead to a polarization of microglia towards a state characterized by the production of inflammatory agents and pro-oxidant species. Sustained amyloid deposition led microglia to a chronic disease-associated state [19]. The transition towards this cell population in CNS tissues seems to be one of the main events causing neuronal death and in turn neurodegeneration. EVs secreted by microglia upon activation showed the presence of inflammatory cytokines along with micro RNAs such as miR-155 able to regulate inflammatory events [20, 21]. EVs originated from activated microglia not only communicate with neurons and astrocytes but also induce an autologous activation towards the pro-inflammatory phenotype of other resting microglial subpopulations enhancing the neuroinflammatory process [22].

Despite the increased knowledge of microglia EVs involvement in the regulation of CNS homeostasis, few studies have focused on EV protein cargo [23–25], and in the Vesiclepedia EV database, only 56 proteins were identified in mouse microglia EVs by a single proteomic study [23].

In this paper, we report a comparative proteome analysis of EVs released by cultured murine immortalized microglia in resting condition or triggered by Aβ with the aim of finding out possible modulations of EV cargo upon amyloid exposure, the pathological event occurring during the development of AD.

## Material and Method

### Cell Culture and EVs Isolation

BV2 cells, an immortalized murine microglial cell line [26], were continuously maintained, from an original gift of prof. Giulio Levi (Istituto Superiore di Sanità, Rome), at the Dipartimento di Scienze Anatomiche, Istologiche, Medico-Legali e dell’Apparato Locomotore. They were grown in Dulbecco’s Modified Eagle Medium (DMEM) supplemented with 10% Fetal Bovine Serum (FBS), 100 U/mL penicillin, 100 μL/mL streptomycin, and 2 mM glutamine at 37 °C in 5% CO_2_/95% humidified air atmosphere. Then they were washed thrice in PBS and posed in a serum-free medium to avoid contamination with bovine exosomes.

BV2 cells were left untreated (NT) or treated (Aβ) with the 25–35 fragment (Aβ_25–35_) of the full-length Aβ amyloid peptide Aβ_1–42_. Prior to use, the synthetic form of Aβ_25–35_ (Bachem) was dissolved to a final concentration of 1 mM in hexafluoroisopropanol (HFIP; Sigma-Aldrich) for its monomerization. HFIP was then removed by evaporation under vacuum, and the peptide was solubilized in DMSO for 10 min. After dilution to 100 μM in DMEM, Aβ_25–35_ was incubated at 4 °C for 24 h for polymerization [27]. Then, it was added to the Aβ sample at a final concentration of 25 μM. After 24 h, cell medium was collected from NT and Aβ cells and protease inhibitors were added.

To assess the inflammatory cell state induced by amyloid treatment, nitrite (NO_2_^−^) levels were determined by using Griess reagent (1 mM sulfanilamide, 1 mM naphthylenediamine dihydrochloride and 100 mM HCl) in culture supernatants of NT and Aβ samples at 24 h. Absorbance was measured at 540 nm, and NO_2_^-^ concentration was determined using sodium nitrite as a standard. Escherichia coli lipopolysaccharide (LPS) (serotype 0127:B8; 0.1 μg/ml) was used as positive control [28].

Cell viability was assessed by measuring lactate dehydrogenase (LDH) released in the culture medium using a cytotoxicity detection kit (Roche, Mannheim, Germany) according to the manufacturer’s protocols [29]. Statistical analyses were conducted using GraphPad Prism version 4.00 software. Data are expressed as means ± SEM. Comparisons were analyzed using *t*test

For each biological replicate, a total of 24 ml cell medium both for NT and Aβ samples, was centrifugated at 300 *g* for 10 min and at 3000 *g* for 30 min to eliminate debris and dead cells. The supernatant was filtered on a 0.22 μm filter to remove EVs over 220 nm size. The filtrate was ultra-centrifuged at 100,000 *g* for 1 h; the pellet was resuspended in 24 ml of Phosphate Buffered Saline pH 7.4 (PBS) and ultra-centrifuged again at 100,000 *g*. The pellet was finally resuspended in 100 μl of modified Laemmli buffer containing 8% Sodium Dodecyl Sulfate (SDS) and 1% n-dodecyl-β-D-maltoside.

### Proteomic Analyses

Protein quantification of cell lysates from NT and Aβ samples was assayed by Protein Assay Dye Reagent (Bio-Rad). To collect similar amount of EV lysates for the following analytical procedures, aliquots were taken relying on protein content as quantified in cell lysates. These aliquots were fractionated by SDS-polyacrylamide gel electrophoresis (SDS-PAGE) on 4–20% Mini-PROTEAN TGX™ gel (Bio-Rad) and stained using a colloidal Coomassie staining.

From each SDS-PAGE lane, twelve slices were excised, and each of them was submitted to a carbamidomethylation treatment and trypsin proteolysis according to Brisdelli et al. [30]. Desalting steps were carried out by solid-phase extraction (SPE) according to Rappsilber et al. [31]. C_18_ reverse-phase loaded Empore™ SPE disks were purchased from Sigma-Aldrich. Peptide mixtures were dried, re-suspended in 50 μL of 0.1% TFA, and analysed by nano-liquid chromatography-tandem mass spectrometry (nano LC-MS/MS). For this purpose, an Ultimate3000 system (Dionex, Sunnyvale, CA, USA) was equipped with a splitting cartridge for nanoflows and connected on-line via a nano-electrospray ion source (Thermo-Fisher Scientific, Waltham, Massachusetts, USA) to an LTQ Orbitrap XL mass spectrometer (Thermo-Fisher Scientific) [30].

Each sample was automatically loaded from the autosampler module of the Ultimate 3000 system at a flow rate of 20 μL/min onto a trap column (Acclaim^®^PepMap^™^ μ-Precolumn, 300 μM × 1 mm, Dionex) in 4% acetonitrile containing 0.1% formic acid. After 4 min, peptides were eluted at 300 nL/min onto a 15 cm column (360 μM OD × 75 μM ID, 15 μM Tip ID; PicoFrit®, New Objective, Woburn, MA, USA), custom packed with a reverse phase (C_18_, 5 μM particle size, 200 Å pore size; Magic C18 AQ, Michrom), by a two-step gradient of solvent B (from 5% to 40% in 120 min, and from 40% to 85% in 15 min). At the end of each run, the eluent was set back to 4% solvent B, and the column was left to equilibrate for 20 min. Eluted peptides were injected and analysed by LTQ Orbitrap XL as in Correani et al. [32]. In particular, tandem mass spectra (MS/MS) were acquired with a data-dependent top-5 method, selecting the five most intense ions with charge states ≥2 detected per survey scan by FTMS if they exceeded an intensity of at least 200 counts. To avoid redundant sequencing of the most abundant peptides, dynamic exclusion was enabled with a repeat count of 1, repeat duration of 30 s, exclusion list size of 300, and exclusion duration of 90 s [32].

Raw files from the nano LC-MS/MS analyses were examined by proteomics software package MaxQuant (version 1.6.0.1, Max Planck Institute of Biochemistry, Martinsried) [33]. The Andromeda search engine was configured for the *Mus musculus* database from UniProtKB (release May 2022, 17102 sequences), including the decoy database of reverse peptides as well as a dataset of commonly detected contaminants in proteomics [32].

The matrix of protein group identification was filtered out by the Perseus software (version 1.6.0.7. Max Planck Institute of Biochemistry) for reverse identifications, contaminants, and peptides “only identified by site”. Protein groups identified with less than one unique peptide and present in only one biological replica were further removed [32].

### Bioinformatic Analysis

Gene Ontology (GO) analyses were performed by the **D**atabase for **A**nnotation, **V**isualization, and **I**ntegrated **D**iscovery (DAVID, v6.8; https//david.ncifcrf.gov/) against the murine genome. The GO analysis of the identified EV proteins was run for cellular localization. The identified proteomes were compared with Vesiclepedia (ex Exocarta) database (http://www.microvesicles.org) using the Venn diagrams webtool (http://bioinformatics.psb.ugent.be/webtools/Venn/).

### Western Blot Analysis

BV2 cells (∼10^6^) were lysed in 100 μL RIPA buffer containing a suitable cocktail of protease inhibitors. The lysates, incubated on ice for 30 min, were then centrifuged at 15,600 *g* for 15 min at 4 °C. Supernatants were subject to protein quantification using a Bradford Assay. Aliquots of cellular lysate and EV samples were separated on 4–12% Bis-Tris Plus Bolt™ (Invitrogen) in MOPS-SDS running buffer and transferred to nitrocellulose membrane (iBlot®2 NC Mini Stacks) using the iBlot®2 system (Invitrogen). After incubation with 5% ECL blocking agent (GE Healthcare Life Sciences) in TBS-T buffer (50 mM Tris, 150 mM NaCl, 0.1% Tween-20, pH 7.5) for 1 h, membranes were challenged with primary rabbit polyclonal antibody anti-CD81 (1:500) (Santa Cruz Biotechnology, sc-9158), rabbit polyclonal antibody anti-CANX/Calnexin (1:500) (BIOSS, bs-1693R), mouse monoclonal anti-Rab11A (1:500) (Santa Cruz Biotechnology, sc-166912) in TBS-T at 4 °C o/n. After three washes with TBS-T, membranes were incubated for 1 h at room temperature with the corresponding horseradish peroxidase-conjugated secondary antibodies: goat anti-mouse IgG-HRP (1:2500) (Santa Cruz Biotechnology, sc-2005) and goat anti-rabbit IgG-HRP (1:2500) (sc-2004, Santa Cruz Biotechnology). Protein signal was visualized by chemiluminescence using ECLTM Prime Western Blotting System (GE Healthcare Life Sciences), detected using a Molecular Imager R_ChemiDoc™, mod. MP System (Bio-Rad Laboratories) and acquired by ImageLab Software (ver. 4.1). Normalization was based on densitometry obtained by Pierce™ Reversible Protein Stain Kit for nitrocellulose membranes (Thermo Scientific). The student’s *t*-test was performed for statistical analyses and differences in protein expression with a *p* value of <0.05 were considered statistically significant.

## Results

**Fig. 1:**
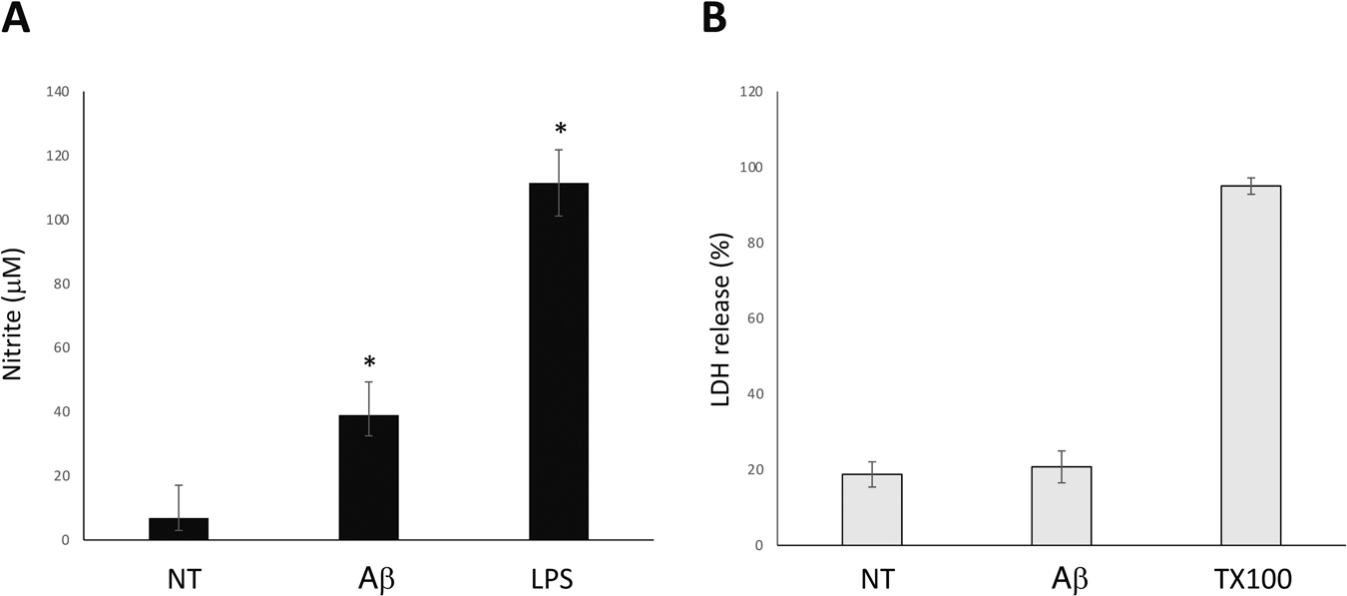
BV2 cells treated with 25 µM Aβ_25–35_ for 24 h were assayed for nitrite production and LDH release. **A** Inflammation was monitored by Griess reaction measuring nitrite levels in culture supernatants. Data from microglial cells treated with LPS (0.1 μg/ml) is shown as a reference. **B** LDH release in the culture medium was assessed for cell viability. Values are represented as the percentage to the maximum value obtained by 2% Triton X-100. *n* = 4; data indicate mean ± SEM. *t*test * *p* < 0.01 versus untreated cultures

The aim of this study was to examine the protein cargo of EVs released by microglia to identify proteins that might be delivered to neuronal and glial compartments. EV protein profiling by a proteomic approach may be useful to understand the regulatory functions of microglia in healthy neural tissue as well as in altered conditions such as inflammatory or phagocytic states occurring in neuronal pathologies and in neurodegeneration. With this aim, we compared proteome profiles of EVs derived from untreated and Aβ-treated microglia. BV2 immortalized murine microglial cells were used as substitutes of primary microglia to obtain an appropriate number of cells suitable for EV proteomic analysis. To stimulate microglia, we used the Aβ_25–35_ peptide which, although is a shorter form compared to the full-length amyloidogenic Aβ_1-42_ peptide, is more manageable and able to induce inflammation [34]. Aβ_25–35_ activity was confirmed by monitoring nitrite release in the medium (Fig. 1A) and it was proven not to be harmful to microglial cell viability (Fig. 1B).

**Fig. 2:**
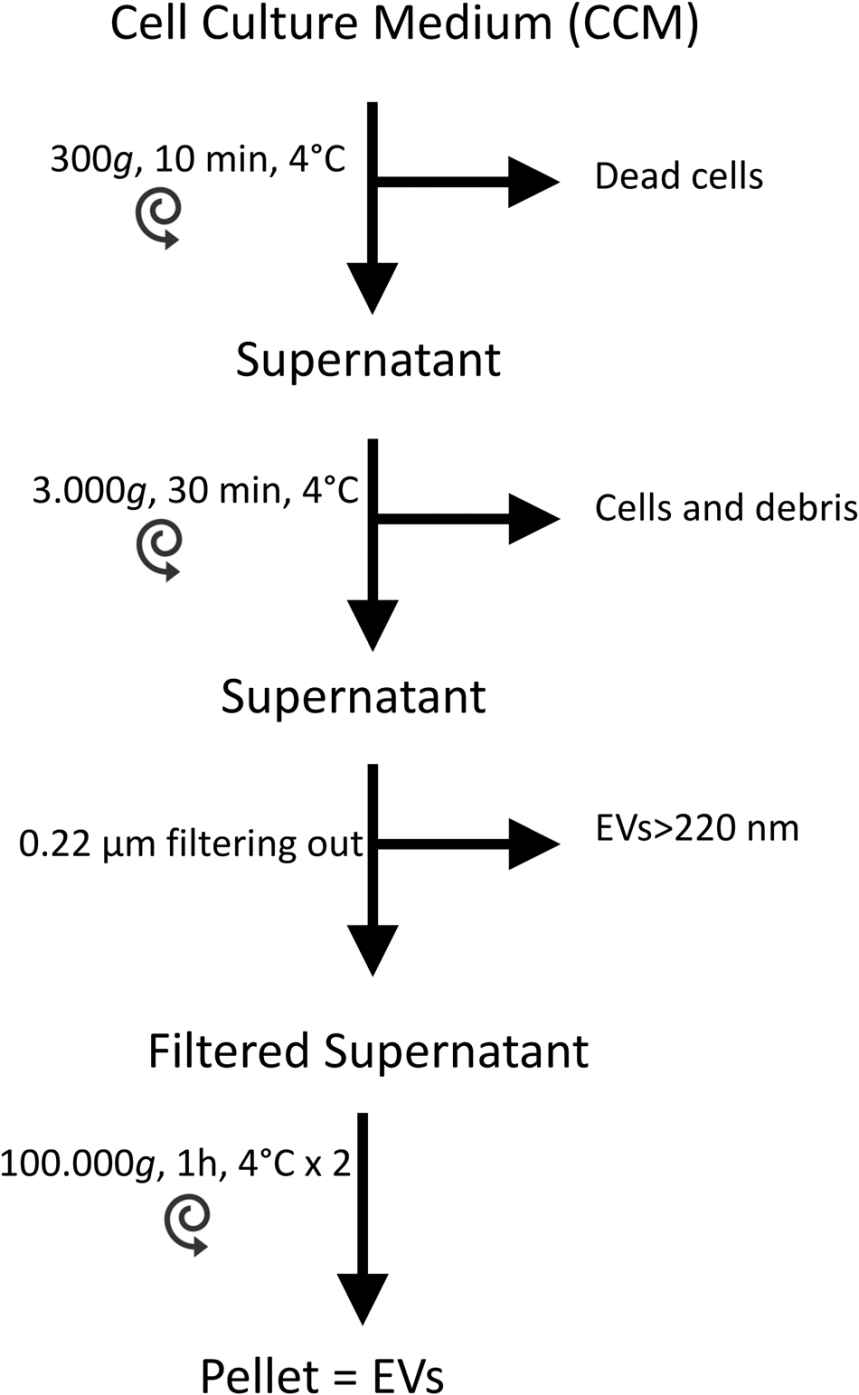
Workflow for EV isolation from untreated and Aβ-treated samples

**Fig. 3:**
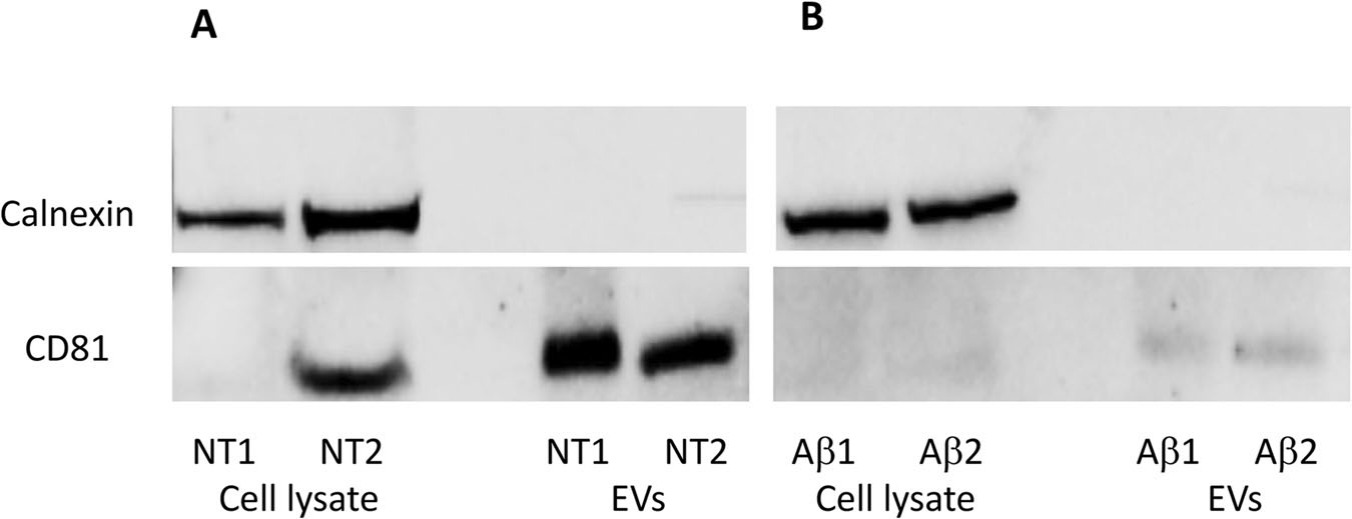
Western blot analyses of CD81 and calnexin on BV2 cell lysates of NT (**A**) and Aβ (**B**) EV samples. Aliquots taken from untreated or treated samples (25 µM Aβ_25–35_) at 24 h, were loaded on a 4–12% SDS-PAGE and immunoblotted using antibodies against CD81 and calnexin

EVs were isolated from cell culture supernatant by serial centrifugations following an established protocol slightly modified [4, 35, 36] (workflow in Fig. 2). To detect possible contaminations of intracellular membranes, EVs obtained from treated and untreated preparations were assayed with antibodies against CD81 and calnexin. The former is an acknowledged EV marker while the latter identifies the ER membrane. Western blot analyses showed positive results for CD81 in both EV preparations from Aβ-treated and untreated cultures (Fig. 3), while no positive outputs were observed for calnexin confirming the prevalent EV origin of our samples.

**Fig. 4:**
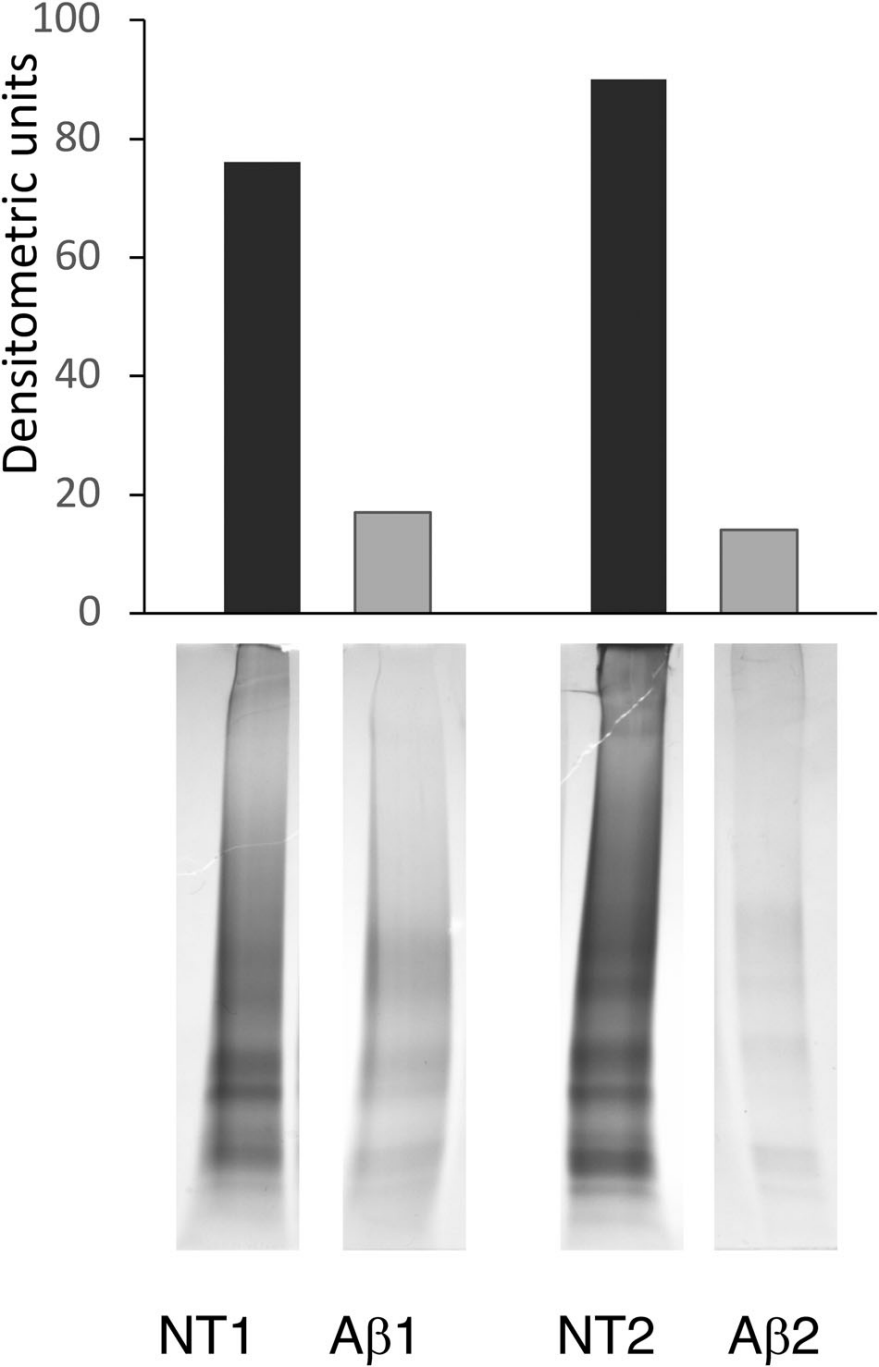
Densitometric analysis of SDS-PAGE of the EV biological replicates from untreated and Aβ-treated samples (NT1/Aβ1 and NT2/Aβ2). Lanes are cropped from SDS-PAGE reported in Fig. 1S

Although a similar amount of proteins was detected in cell lysates from both Aβ -treated and untreated cells, we observed that the yield of EV proteins was quite different, with the EVs from the Aβ-treated sample showing a dramatic lower amount (81% minus) in protein content with respect to those from the untreated one (Figs. 4 and 1S).

**Fig. 5:**
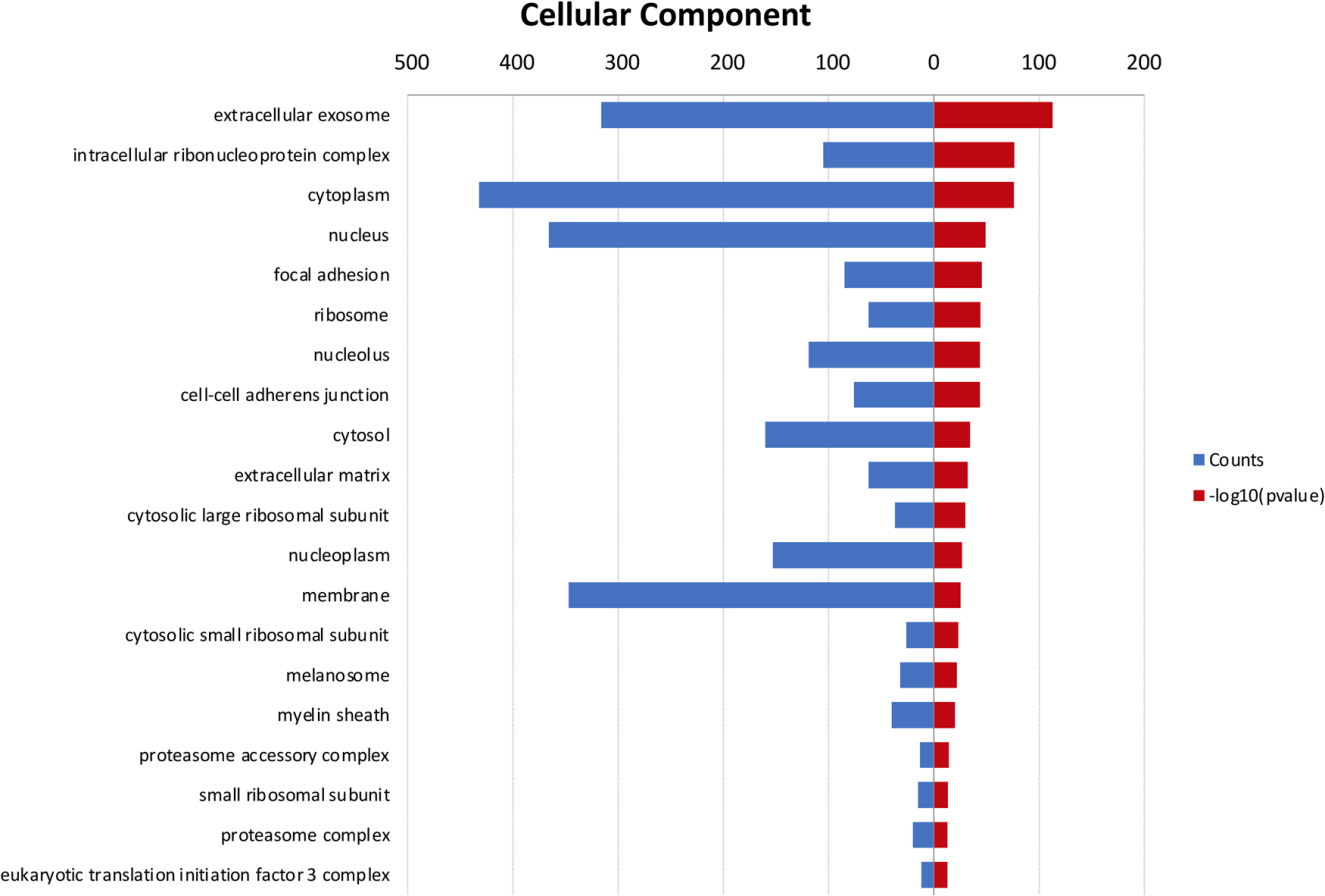
Gene Ontology (GO) analysis of cellular component of the 642 identified proteins shared by untreated BV2–derived EV samples

**Fig. 6:**
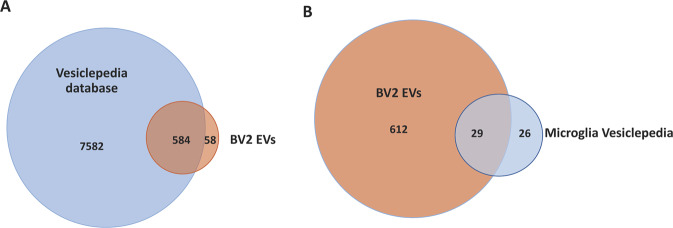
Comparison of protein hits assigned to the microglia vesicle proteome in this study (red circle) against total Vesiclepedia database (**A**) (blue circle) and BV2 microglia Vesiclepedia database (**B**) (blue circle)

Raw mass spectrometric data of the two untreated biological replicates NT1 and NT2, analysed by the MaxQuant proteomic platform, led to the identification of 642 proteins shared between the two samples. The complete list of these proteins is reported in Table S1. The Gene ontology of this list based on David Bioinformatics Resource 6.8 is depicted in Fig. 5 and showed that the most enriched term of the cellular component was assigned to the extracellular exosome compartments. Moreover, as further validation of the success of our EV isolation strategy, a comparison of the identified 642 proteins with all the Vesiclepedia Database entries showed that 91% of them (584 out of 642) were already localized in EVs (Fig. 6A). More specifically, when we compared our proteomic result with the subset of Vesiclepedia Database comprising 56 proteins previously found in exosomes from mouse microglial N9 cultures [23], we found 29 out of 56 overlapping proteins yielding a consequent sharing value of 52% (Fig. 6B and Table 1).

**Table 1:** Identified proteins in resting microglia EVs shared with Microglia Vesiclepedia entries

Protein	Gene Name
Fructose-bisphosphate aldolase	ALDOA
Aminopeptidase	ANPEP
Annexin A2	ANXA2
Macrophage-capping protein	CAPG
CD14 antigen	CD14
CD81 antigen	CD81
**Cofilin 1**	**CFL1**
Eukaryotic translation initiation factor 3 subunit F	EIF3F
Enolase 1	ENO1
**High affinity immunoglobulin epsilon receptor subunit gamma**	**FCER1G**
Glyceraldehyde-3-hosphate dehydrogenase	GAPDH
Guanine nucleotide-binding protein G(i) subunit alpha-2	GNAI2
Histone H2B type 1-P	HIST1H2BP
Heat shock protein HSP 90-alpha	HSP90AA1
Heat shock 70 kDa protein 8	HSPA8
Ras GTPase-activating-like protein IQGAP1	IQGAP1
**Integrin beta-2**	**ITGB2**
**Galectin-3**	**LGALS3**
**Lipoprotein lipase**	**LPL**
**Moesin**	**MSN**
Myosin-9	MYH9
Programmed cell death 6 interacting protein	PDCD6IP
Phosphoglycerate mutase 1	PGAM1
Phosphoglycerate kinase 1	PGK1
Pyruvate kinase muscle isozyme	PKM
**Ras-related protein Rab11A**	**RAB11A**
**Ras-related protein Rab7**	**RAB7**
**Syndecan-binding protein**	**SDCBP**
**Solute carrier family 16 member 1**	**SLC16A1**

Entries of proteins undetected in the EVs derived from Aβ samples are in bold

**Fig. 7:**
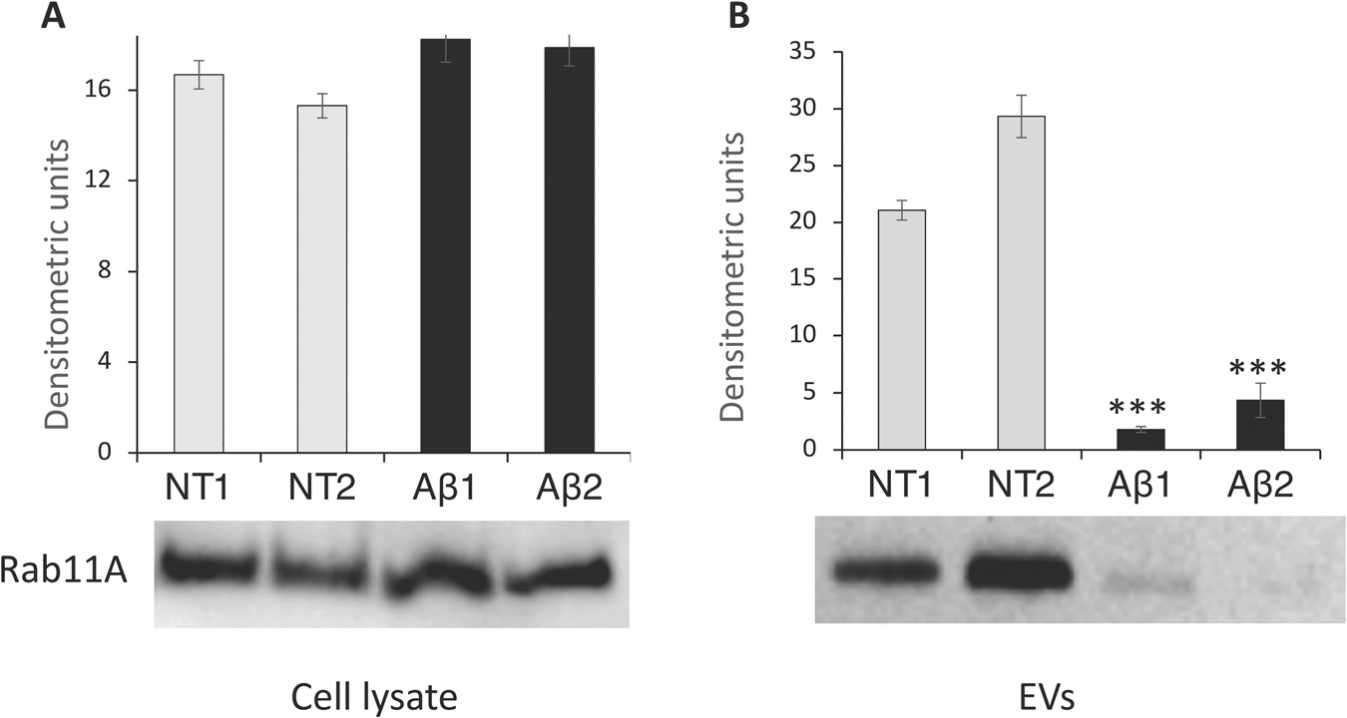
Western blot analyses of Rab11A on BV2 cell lysate (**A**) and EVs (**B**). Aliquots taken from untreated or treated samples with 25 µM Aβ_25–35_ for 24 h, were loaded to 4–12% SDS-PAGE and immunoblotted with anti-Rab11A antibody. Densitometric analyses performed with ImageJ software were normalized on total densitometric absorbance. Light gray bar = untreated cells (NT); black bar = treated cells (Aβ). Reported results are the mean of three experiments ± SEM. ^***^p < 0.001 Aβ1 *vs*. NT1 and Aβ2 vs. NT2

As mentioned above, a striking feature of the EV samples obtained from the two Aβ-treated biological replicates, Aβ1 and Aβ2, was their lower protein content compared to that of EVs from untreated samples. We identified 210 proteins shared by Aβ1 and Aβ2 biological replicates. All these proteins were already present in the list of 642 identified proteins shared by NT1 and NT2. Comparing the list of proteins from Aβ samples with the 29 proteins shared by NTs and microglia Vesiclepedia entries (Table 1), we found that 10 out of those 29 proteins were undetected in the Aβ sample and their entries are highlighted in Table 1. To assess whether the absence of these proteins was due to a general decrease of their cellular expression due to Aβ stimulation, we queried a database we set up in a previously published work, derived from the same cellular model used in this study [32]. This database consisted of a list of mass spectrometric identified proteins from resting and Aβ-treated BV2 samples in the plasma membrane compartment. Relative quantification of the identified proteins was achieved using a Stable Isotope Metabolic Labelling of Amino acid residues in Cell cultures approach followed by nano LC-MS/MS analysis. The quantitative ratio for the 10 proteins highlighted in Table 1 showed that they were equally represented in the plasma membrane compartment of both Aβ-treated and untreated samples (Table 2). Results seemed to indicate that the disappearance of these proteins in the EV proteome of Aβ-treated samples was not determined by a generally reduced level of their expression. We validated these data by focusing on Rab11A protein, a key factor in the recycling route of amyloid species. By western blot analysis, we confirmed the presence of a similar quantity of Rab11A in whole lysates from Aβ-treated and untreated cells (Fig. 7A). Conversely, the same analysis performed on EV samples, normalized for the total amount of protein loaded, revealed a net decrease of Rab11A in the EVs from Aβ-treated samples with respect to the EVs from resting cells (Fig. 7B). Taken together these results point to a general reduction of Rab11A in the EVs derived from Aβ-treated microglia due to either a lower general production of EVs and/or to a specific lower loading of this protein in EVs with respect to untreated microglia.

**Table 2:** Quantitative ratio determined in plasma membrane of Aβ treated and untreated microglia for protein undetected in EVs from Aβ samples

Proteins	Aβ/NT ratio^a^
Cofilin 1	1,0
High affinity immunoglobulin epsilon receptor subunit gamma	1,0
Integrin beta-2	0,9
Galectin-3	0,8
Lipoprotein lipase	NI
Moesin	1,0
Ras-related protein Rab11A	0,9
Ras-related protein Rab7	0,9
Syndecan-binding protein	1,0
Solute carrier family 16 member 1	1,1

^a^Data from reference [29]; NI, not identified

## Discussion

The central role of microglia in the CNS in defending and remodeling the neuronal population has prompted, in the last decades, a great effort of research designed to disclose the principal mechanisms responsible for the effective impact of these cells. This interest was also aimed to unveil the involvement of microglia in many neurological and neurodegenerative diseases. Recently, EVs produced by microglia have been highlighted as important tools in maintaining brain homeostasis as well as a potential agents for sustaining and developing neurodegenerative diseases. Investigation of the protein content of microglial EVs has been recently attempted in resting [23] and LPS-activated states [25]. In this paper, we aimed to contribute to this effort by investigating the protein content of EVs secreted by microglia in resting and in a cellular model of AD challenging microglial cells with Aβ. For this purpose, murine BV2 cells and the synthetic amyloid peptide Aβ_25–35_ were chosen to produce stable replicates. This task was accomplished as proved by the fact that a great number of identified proteins were shared in both biological replicates of resting samples. This list of proteins identified in EVs derived from resting microglial cells was greater than the one reported in the murine microglial Vesciclepedia database where 56 entries specifically refer to EVs derived from an N9 immortalized microglial cell line [23].

Two other similar proteomic data, not yet included in Vesiclepedia, have been recently published. One of them was a study on leech microglial EV proteome where the total number of proteins identified was 776, close to our finding for the EVs derived from untreated microglia [24]. A more restricted number of proteins was found in another study aimed to compare the EV content in resting and LPS-activated microglia [25]. Many factors should be considered to account for these variabilities such as differences in the methodological setup as the dissimilar extraction methodologies, the type of biological organism, the different proteomic platforms, and data filtering. All these items may explain the discrepant quantitative yield obtained among these different studies.

Our list of proteins matched well with the total Vesiclepedia database and with the proteins specifically found in mouse microglia. Gene ontology analysis confirmed these data showing exosome localization as the most enriched compartment.

Treatment of microglia with Aβ produced a drop in the total number of proteins identified in EV samples. All these proteins were already present in the list of proteins of the resting BV2 EV proteome. This result seems to indicate that, after 24 h of amyloid treatment, there was not any detectable expression of novel proteins in EVs proteome suggesting that the Aβ amyloid challenge does not reshape the quality of protein expression but rather affects the quantity of delivered protein by EVs during the settlement of the chronic microglial phenotype. An opposite behavior was observed in BV2 cells treated with LPS, a pro-inflammatory agent, and EVs were collected after 12 h [25]. Proteomes from resting and LPS-treated microglia EVs shared only a restricted number of proteins showing that a dramatic qualitative change occurred in EVs during the transition from the resting form towards the M1 pro-inflammatory phenotype. These observations seem to confirm that the M1 phenotype is unlikely to overlap with the one evoked by the prolonged treatment of microglia with Aβ as it is emerging from several studies aimed to comprehend microglia state in neurodegenerative diseases [37–39].

Among proteins identified in the resting microglia in both our study and the previous one reported in Vesciclepedia [23], ten proteins were missed in the Aβ-treated sample and most of them were localized in the plasma membrane compartment such as Cofilin-1, Syndecan binding protein, Rab11A, and Rab7. Quantitative mass spectrometric analyses of these proteins in the plasma membrane of Aβ-treated and untreated microglia showed no significant change between them. This result ruled out that the drop of these proteins in EVs secreted from Aβ-treated samples might be due to a lower expression of these proteins in the Aβ-treated cells compared to the untreated control. Although these proteins are, to various degrees, involved in the molecular events occurring in the endosomal/exosomal route [40–42], Rab11A has received particular attention for its involvement in the exosomal recycling from the multivesicular late endocytic compartments to the plasma membrane [43]. Focusing on this protein, we observed that Rab11A was less represented in Aβ-treated microglia EV cargo with respect to the EVs from the untreated samples. Rab11A belongs to a subfamily of the small Rab GTPase family and functions as a cellular switch depending on GDP/GTP bound state [44]. Many lines of evidence demonstrated that only a few proteins of the subfamily (e.g., Rab11A, Rab27 and Rab35) showed a direct and significant involvement in the biogenesis and secretion of exosomes. Furthermore, Rab11A has been associated with neurodegenerative pathologies like Parkinson’s and Alzheimer’s diseases featuring as an important regulator for the trafficking of both alfa-synuclein [45] or Aβ [46] arising during the development of these diseases. Rab11A is responsible for the fusion and docking of multivesicular endosomes containing the misfolded proteins to the plasma membrane. This process is essential for exosome release and may help neurons to clear out amyloid species from inside cells to avoid engulfing lysosome machinery [47]. On the other hand, it was demonstrated that a lack of expression of Rab11A may counteract the settling of tissue AD phenotypes by lowering pathological EV cargo traffic that favors the widespread of toxic species [48, 49]. Loss of Rab11A expression in neurons decreases also intracellular Aβ by downregulation of its endocytosis and of axon localization of BACE1, the beta-secretase that along with gamma-secretase is responsible for the cleavage of APP to produce Aβ species [49–51]. Microglial EV Rab11A delivered to the neurons may be essential for the physiological recycling and secretion of the Aβ species while, in the pathological state, the observed decrease of Rab11A in EVs may break the balance between these processes and thus be harmful to neurons increasing their intracellular amyloid toxic burden and eventually leading to neuronal death.

## Conclusions

In this paper, we extended the number of proteins identified in the resting microglia EVs using BV2 cells. We also highlighted that Aβ-treatment induces in microglia a decrease in EV protein cargo and a lower loading in the EV cargo of Rab11A which is considered a key factor in the recycling routes of amyloid species. We tentatively speculate that these alterations we observed in Aβ-treated microglia may represent molecular events that mark the “disease-associated microglia phenotype”. This subset of the microglia population, unique for transcriptional and functional signatures, has been recently proposed as a major factor in brain diseases and more specifically in neurodegenerative pathologies [19].

## Supplementary Information

Supplementary Information

Supplementary Information

Supplementary Information

## Abbreviations

EVs: Extracellular vesicles
AD: Alzheimer’s disease
Aβ: beta-amyloid peptides
MVs: Microvesicles
CNS: Central Nervous System
DMEM: Dulbecco’s Modified Eagle Medium
FBS: Fetal Bovine Serum
HFIP: Hexafluoroisopropanol
LPS: Lipopolysaccharide
LDH: Lactate dehydrogenase
PBS: Phosphate Buffered Saline pH 7.4
SDS: Sodium Dodecyl Sulfate
SDS-PAGE: SDS-polyacrylamide gel electrophoresis
SPE: Solid Phase Extraction
LC-MS/MS: Liquid chromatography tandem mass spectrometry
GO: Gene Ontology
DAVID: Database of Annotation, Visualization and Integrated Discovery
RIPA: Radioimmune Precipitation Assay buffer
MOPS: 3-(N-morpholino) propanesulfonic acid
TBS: TRIS Buffered Saline
