# Supplementary material for: Rab11A Depletion in Microglia-Derived Extracellular Vesicle Proteome upon Beta-Amyloid Treatment
Source: Cell Biochem Biophys. 2023 Mar 30;81(2):337–47. doi: 10.1007/s12013-023-01133-4 (PMC10257621; doi:10.1007/s12013-023-01133-4)
Supplement: Supplementary file 2 — Supplementary Information [file 12013_2023_1133_MOESM2_ESM.pdf]

Table S1

Protein IDs[a]	Majority protein Ids[b]	Protein names	Gene names	Peptides Aβ1[c]	Peptides Aβ2[c]	Peptides NT1[c]	Peptides NT2[c]	Unique peptides Aβ1[d]	Unique peptides Aβ2[d]	Unique peptides NT1[d]	Unique peptides NT2[d]	Sequence coverage [%][e]	Mol. weight [kDa][f]	Intensity Aβ1[g]	Intensity Aβ2[g]	Intensity NT1[g]	Intensity NT2[g]	Q-value[h]	Score[i]
Q9D2R0	Q9D2R0	Acetoacetyl-CoA synthetase	Aacs	1	0	1	3	1	0	1	3	4,3	75,199	437780	0	190620	2435300	0	17,673
Q8BGQ7	Q8BGQ7	Alanine--tRNA ligase, cytoplasmic	Aars	4	0	4	12	4	0	4	12	20	106,91	1,11E+07	0	1,98E+07	5,24E+07	0	323,31
P61222	P61222	ATP-binding cassette sub-family E member 1	Abce1	1	0	2	7	1	0	2	7	18,4	67,314	4600100	0	3882800	2,00E+07	0	132,94
Q6P542	Q6P542	ATP-binding cassette sub-family F member 1	Abcf1	2	0	2	7	2	0	2	7	14,1	94,944	1103400	0	1,24E+07	3,24E+07	0	285,66
Q921H8;Q8VCH0	Q921H8;Q8VCH0	3-ketoacyl-CoA thiolase A, peroxisomal;3-ketoacyl-CoA thiolase B, peroxisomal	Acaa1a;Acaa1b	1	0	1	5	1	0	1	5	21,5	43,953	161520	0	148140	5648500	0	84,631
Q5SWU9	Q5SWU9	Acetyl-CoA carboxylase 1;Biotin carboxylase	Acaca	2	0	7	9	1	0	5	6	6,5	265,25	1940500	0	1,40E+07	1,00E+07	0	71,425
Q8CAY6	Q8CAY6	Acetyl-CoA acetyltransferase, cytosolic	Acat2	1	0	1	2	1	0	1	2	11,1	41,297	320440	0	355990	1312800	0	22,33
Q91V92	Q91V92	ATP-citrate synthase	Acly	3	0	4	15	3	0	4	15	21,2	119,73	2036600	0	1,23E+07	3,68E+07	0	116,46
Q9D358	Q9D358	Low molecular weight phosphotyrosine protein phosphatase	Acp1	1	1	2	2	1	1	2	2	31,6	18,192	1263800	58151	3973800	628590	0	38,93
Q9QUJ7;Q9CZW4	Q9QUJ7	Long-chain-fatty-acid--CoA ligase 4	Acsl4	1	0	1	15	1	0	1	15	27	79,076	1362100	0	3124900	8,44E+07	0	184,27
P60710	P60710	Actin, cytoplasmic 1;Actin, cytoplasmic 1, N-terminally processed	Actb	12	5	14	18	1	1	1	1	61,9	41,736	1,29E+08	8044300	7,30E+08	8,52E+08	0	323,31
Q8BFZ3	Q8BFZ3	Beta-actin-like protein 2	Actbl2	3	1	4	5	1	0	1	1	20,7	42,004	2679100	0	1,58E+07	1,14E+07	0,00091743	6,7108
P63260	P63260	Actin, cytoplasmic 2;Actin, cytoplasmic 2, N-terminally processed	Actg1	11	4	14	18	0	0	1	1	61,9	41,792	0	0	2399300	3108400	0	62,805
Q9Z2N8	Q9Z2N8	Actin-like protein 6A	Actl6a	0	0	1	1	0	0	1	1	6,3	47,447	0	0	416810	531360	0	12,837
P61161	P61161	Actin-related protein 2	Actr2	1	0	2	6	1	0	2	6	20,6	44,76	135310	0	3671200	2,46E+07	0	93,177
Q99JY9;Q641P0	Q99JY9	Actin-related protein 3	Actr3	3	0	3	9	3	0	3	9	23	47,357	3482800	0	1,42E+07	2,45E+07	0	87,212
P54923	P54923	[Protein ADP-ribosylarginine] hydrolase	Adprh	0	0	1	1	0	0	1	1	7,5	40,068	0	0	517240	251720	0	13,093
Q9JKV1	Q9JKV1	Proteasomal ubiquitin receptor ADRM1	Adrm1	1	0	1	1	1	0	1	1	3,7	42,06	450170	0	1476400	1882700	0,0017376	6,4387
P54822	P54822	Adenylosuccinate lyase	Adsl	2	2	2	4	2	2	2	4	9,1	54,866	4773200	452500	1,75E+07	1,33E+07	0	103,44
P46664	P46664	Adenylosuccinate synthetase isozyme 2	Adss	0	0	1	1	0	0	1	1	7,7	50,02	0	0	486210	1420900	0	34,104
P31230	P31230	Aminoacyl tRNA synthase complex-interacting multifunctional protein 1;Endothelial monocyte-activating polypeptide 2	Aimp1	1	0	2	4	1	0	2	4	29	33,997	744000	0	2671500	8064600	0	92,818
Q8R010	Q8R010	Aminoacyl tRNA synthase complex-interacting multifunctional protein 2	Aimp2	0	0	2	4	0	0	2	4	20,9	35,377	0	0	2035000	8678200	0	51,835
Q9JII6	Q9JII6	Alcohol dehydrogenase [NADP(+)]	Akr1a1	2	1	2	5	2	1	2	5	17,8	36,586	1320000	110310	2930400	6434800	0	36,027
P45376	P45376	Aldose reductase	Akr1b1	1	1	3	4	1	1	3	4	22,8	35,732	213340	52295	1,31E+07	9523400	0	74,458
Q571I9	Q571I9	Aldehyde dehydrogenase family 16 member A1	Aldh16a1	1	0	1	3	1	0	1	3	5,4	84,755	818970	0	1228900	4599400	0	102,63
Q9JLJ2	Q9JLJ2	4-trimethylaminobutyraldehyde dehydrogenase	Aldh9a1	3	0	3	5	3	0	3	5	16,8	53,514	2278500	0	2,15E+07	8494800	0	137,69
P05064	P05064	Fructose-bisphosphate aldolase A	Aldoa	1	0	2	11	1	0	2	11	40,1	39,355	1026200	0	3291300	2,79E+07	0	185,23
Q99NH0	Q99NH0	Ankyrin repeat domain-containing protein 17	Ankrd17	0	0	1	2	0	0	1	2	1,5	274,21	0	0	153130	876310	0	19,579
Q9EST5	Q9EST5	Acidic leucine-rich nuclear phosphoprotein 32 family member B	Anp32b	1	0	2	2	1	0	2	2	7,7	31,078	137940	0	6035000	272500	0	12,331
P97449	P97449	Aminopeptidase N	Anpep	1	0	2	6	1	0	2	6	8,6	109,65	145250	0	695960	2,04E+07	0	71,16
P10107	P10107	Annexin A1	Anxa1	2	0	1	9	2	0	1	9	37	38,734	208050	0	455820	6361300	0	155,3
P97384	P97384	Annexin A11	Anxa11	1	1	2	7	1	1	2	7	19,7	54,079	464980	199430	2919200	7781900	0	54,086
P07356	P07356	Annexin A2	Anxa2	3	1	10	15	3	1	10	15	41,6	38,676	1551000	620410	2,38E+07	2,13E+07	0	323,31
P97429	P97429	Annexin A4	Anxa4	5	3	7	10	5	3	7	10	37,9	35,915	2660400	3313000	1,78E+07	1,32E+07	0	323,31
P48036	P48036	Annexin A5	Anxa5	3	4	8	6	3	4	8	6	33,2	35,752	5343100	3021600	7,31E+07	1,16E+07	0	103,22
Q07076	Q07076	Annexin A7	Anxa7	1	1	2	4	1	1	2	4	10,6	49,925	1464500	249760	904790	6546900	0	52,741
P22892	P22892	AP-1 complex subunit gamma-1	Ap1g1	1	0	1	1	1	0	1	1	1,1	91,349	694590	0	659900	952510	0,0033698	6,3421
P84091	P84091	AP-2 complex subunit mu	Ap2m1	0	0	1	3	0	0	1	3	8	49,654	0	0	76592	1,01E+07	0	19,367
P28352	P28352	DNA-(apurinic or apyrimidinic site) lyase;DNA-(apurinic or apyrimidinic site) lyase, mitochondrial	Apex1	0	0	1	3	0	0	1	3	16,7	35,49	0	0	586350	1126600	0	26,008
Q99J72	Q99J72	DNA dC->dU-editing enzyme APOBEC-3	Apobec3	1	0	1	4	1	0	1	4	9,8	52,213	209660	0	1694200	5067500	0	109,29
P08226	P08226	Apolipoprotein E	Apoe	6	3	7	9	6	3	7	9	33,4	35,866	1,71E+07	837070	1,09E+07	6,86E+07	0	238,44
P08030	P08030	Adenine phosphoribosyltransferase	Aprt	2	0	5	5	2	0	5	5	47,8	19,724	776200	0	4,45E+07	7825200	0	43,157
8BSL7;P84078;P61205;P840	Q8BSL7;P84078;P61205	ADP-ribosylation factor 2;ADP-ribosylation factor 1;ADP-ribosylation factor 3	Arf2;Arf1;Arf3	2	0	2	3	1	0	1	2	26,5	20,746	2047000	0	896080	4393800	0	45,291
Q99PT1	Q99PT1	Rho GDP-dissociation inhibitor 1	Arhgdia	1	0	1	4	1	0	1	4	30,4	23,407	598870	0	1797500	7675500	0	34,895
Q9JM76	Q9JM76	Actin-related protein 2/3 complex subunit 3	Arpc3	1	0	2	1	1	0	2	1	10,1	20,524	837810	0	9183100	879690	0	12,286
P59999	P59999	Actin-related protein 2/3 complex subunit 4	Arpc4	2	0	3	2	2	0	3	2	19	19,667	2599100	0	3716900	990270	0	55,08
Q61024	Q61024	Asparagine synthetase [glutamine-hydrolyzing]	Asns	1	0	2	5	1	0	2	5	12,3	64,282	961130	0	1,28E+07	1,36E+07	0	51,152
Q9D906	Q9D906	Ubiquitin-like modifier-activating enzyme ATG7	Atg7	0	0	1	2	0	0	1	2	6,2	77,519	0	0	1012200	655540	0	18,393
Q9CWJ9	Q9CWJ9	Bifunctional purine biosynthesis protein PURH;Phosphoribosylaminoimidazolecarboxamide formyltransferase;IMP cyclohydrolase	Atic	8	4	10	16	8	4	10	16	46,5	64,217	1,20E+07	926940	6,73E+07	7,28E+07	0	290,6
VDN2;Q9WV27;Q9Z1W8;Q64	Q8VDN2	Sodium/potassium-transporting ATPase subunit alpha-1	Atp1a1	6	2	7	27	4	2	5	15	30,5	112,98	1,23E+07	226370	3,80E+07	3,58E+08	0	323,31
Q6PIC6;Q6PIE5	Q6PIC6;Q6PIE5	Sodium/potassium-transporting ATPase subunit alpha-3;Sodium/potassium-transporting ATPase subunit alpha-2	Atp1a3;Atp1a2	3	0	3	17	1	0	1	5	20,6	111,69	49868	0	5599600	1,44E+07	0	249,01
P97370	P97370	Sodium/potassium-transporting ATPase subunit beta-3	Atp1b3	1	0	1	6	1	0	1	6	24,5	31,775	112610	0	138700	2,33E+07	0	60,464
G5E829	G5E829	Plasma membrane calcium-transporting ATPase 1	Atp2b1	1	0	2	15	1	0	2	8	17	134,75	208420	0	2605700	3,74E+07	0	240,08
Q03265	Q03265	ATP synthase subunit alpha, mitochondrial	Atp5a1	0	0	1	1	0	0	1	1	5,2	59,752	0	0	6514900	869360	0	13,792
Q9CYN9	Q9CYN9	Renin receptor	Atp6ap2	1	1	1	3	1	1	1	3	8,6	39,091	71461	516670	1590000	2177200	0	19,098
P50516	P50516	V-type proton ATPase catalytic subunit A	Atp6v1a	4	1	5	12	4	1	5	12	27,1	68,325	1,96E+07	441820	3,23E+07	4,35E+07	0	323,31
P62814	P62814	V-type proton ATPase subunit B, brain isoform	Atp6v1b2	3	1	3	11	3	1	3	11	23,7	56,55	4023200	83986	8501800	3,93E+07	0	245,28
P50518	P50518	V-type proton ATPase subunit E 1	Atp6v1e1	1	0	1	3	1	0	1	3	14,6	26,157	261880	0	1468200	6712300	0	32,083
P28658	P28658	Ataxin-10	Atxn10	1	0	3	3	1	0	3	3	14,5	53,706	369770	0	2387800	3515700	0	277,57
Q9Z1R2	Q9Z1R2	Large proline-rich protein BAG6	Bag6	1	0	2	1	1	0	2	1	4,9	121,04	0	0	1813400	1106100	0	20,593
Q9CY64	Q9CY64	Biliverdin reductase A	Blvra	3	1	5	8	3	1	5	8	29,8	33,524	5589700	600970	2,01E+07	1,35E+07	0	66,441
P97452	P97452	Ribosome biogenesis protein BOP1	Bop1	0	0	1	1	0	0	1	1	2,7	82,545	0	0	444310	347500	0	11,475
Q8C3R1	Q8C3R1	BRCA1-associated ATM activator 1	Brat1	0	0	1	1	0	0	1	1	1,6	89,085	0	0	405350	238080	0,00095238	7,113
P18572	P18572	Basigin	Bsg	1	1	2	5	1	1	2	5	16,7	42,444	1595900	549160	7967700	2,98E+07	0	174,01
Q9WVA3	Q9WVA3	Mitotic checkpoint protein BUB3	Bub3	0	0	1	4	0	0	1	4	15,6	36,954	0	0	487680	4852200	0	30,709

B2RQC6	B2RQC6	CAD protein;Glutamine-dependent carbamoyl-phosphate synthase;Aspartate carbamoyltransferase;Dihydroorotase	Cad	1	0	11	10	1	0	11	10	8,2	243,24	348020	0	1,88E+07	1,08E+07	0	146,69
P24452	P24452	Macrophage-capping protein	Capg	2	1	3	3	2	1	3	3	13,1	39,24	3156300	345460	7378300	7106200	0	32,779
P47753	P47753	F-actin-capping protein subunit alpha-1	Capza1	2	1	2	3	1	0	1	2	13,6	32,939	741790	0	319420	5097000	0	15,835
P47754	P47754	F-actin-capping protein subunit alpha-2	Capza2	2	1	2	6	1	0	1	5	32,2	32,967	1,02E+07	89769	1,95E+07	1,66E+07	0	194,6
P47757	P47757	F-actin-capping protein subunit beta	Capzb	0	0	1	10	0	0	1	10	43,3	31,345	0	0	291670	1,95E+07	0	84,21
P48758;Q8K354	P48758	Carbonyl reductase [NADPH] 1	Cbr1	2	0	3	4	2	0	3	4	17	30,641	1042700	0	2299700	4289500	0	26,024
Q9JIG7	Q9JIG7	Coiled-coil domain-containing protein 22	Ccdc22	4	0	4	4	4	0	4	4	11,6	70,843	2855200	0	6808200	4044000	0	48,931
P80314	P80314	T-complex protein 1 subunit beta	Cct2	10	2	12	14	10	2	12	14	46,2	57,477	3,76E+07	3675400	1,88E+08	3,45E+07	0	323,31
P80318	P80318	T-complex protein 1 subunit gamma	Cct3	12	2	15	18	12	2	15	18	47,9	60,629	1,17E+08	9610400	2,23E+08	9,69E+07	0	323,31
P80315	P80315	T-complex protein 1 subunit delta	Cct4	10	3	11	20	10	3	11	20	47,3	58,066	2,91E+07	2241100	1,18E+08	7,97E+07	0	323,31
P80316	P80316	T-complex protein 1 subunit epsilon	Cct5	4	1	5	11	4	1	5	11	31,8	59,623	1,74E+07	92955	7,06E+07	2,95E+07	0	154,36
P80317;Q61390	P80317	T-complex protein 1 subunit zeta	Cct6a	3	1	4	9	3	1	4	9	30,3	58,004	8516900	2142900	5,15E+07	3,75E+07	0	135,12
P80313	P80313	T-complex protein 1 subunit eta	Cct7	6	4	7	13	6	4	7	13	34	59,652	2,05E+07	872610	4,14E+07	3,82E+07	0	217,6
P42932	P42932	T-complex protein 1 subunit theta	Cct8	9	3	8	11	9	3	8	11	27,7	59,555	2,19E+07	1393800	6,07E+07	2,38E+07	0	109,02
P10810	P10810	Monocyte differentiation antigen CD14	Cd14	2	2	3	6	2	2	3	6	21,9	39,203	1417600	475410	3616700	1,24E+07	0	173,25
Q76KJ5	Q76KJ5	DNA-directed RNA polymerase I subunit RPA34	Cd3eap	0	0	1	2	0	0	1	2	12,3	43,082	0	0	1783700	449840	0	17,645
P15379	P15379	CD44 antigen	Cd44	0	0	1	2	0	0	1	2	2,7	85,616	0	0	9738900	1,80E+07	0	12,448
P11440	P11440	Cyclin-dependent kinase 1	Cdk1	2	1	3	4	1	1	2	3	22,2	34,106	153720	107180	1,06E+07	8432100	0	44,646
9J95;Q3V3A1;Q04899;O3549	9J95;Q3V3A1;Q04899;O3549	Cyclin-dependent kinase 6;Cyclin-dependent kinase 3;Cyclin-dependent kinase 4;Cyclin-dependent kinase 2;Cyclin-dependent kinase 9;Cyclin-dependent kinase 15;Cyclin-dependent kinase 18;Cyclin-dependent kinase 14;Cyclin-dependent kinase 16;Cyclin-dependent kinase 17;Cyclin-dependent kinase 12;Cyclin-dependent kinase 13	;Cdk15;Cdk18;Cdk14	2	0	2	2	1	0	1	1	6,1	37,028	334280	0	643350	329730	0,001773	6,5115
P18760;P45591	P18760	Cofilin-1	Cfl1	2	1	1	5	2	1	1	5	27,1	18,559	477690	79015	628170	1,05E+07	0	71,105
Q6PDQ2;A2A8L1	Q6PDQ2;A2A8L1	Chromodomain-helicase-DNA-binding protein 4;Chromodomain-helicase-DNA-binding protein 5	Chd4;Chd5	1	0	1	3	1	0	1	3	2	217,75	474940	0	2491600	2065700	0	22,305
A2AGT5	A2AGT5	Cytoskeleton-associated protein 5	Ckap5	0	0	1	2	0	0	1	2	1,9	225,63	0	0	488240	622300	0	17,069
Q9Z1Q5	Q9Z1Q5	Chloride intracellular channel protein 1	Clic1	2	0	2	1	2	0	2	1	15,8	27,013	387830	0	3809000	1669000	0	20,248
Q68FD5	Q68FD5	Clathrin heavy chain 1	Cltc	13	4	32	60	13	4	32	60	37,5	191,55	5,09E+07	3166900	2,32E+08	8,62E+08	0	323,31
Q06890	Q06890	Clusterin;Clusterin beta chain;Clusterin alpha chain	Clu	1	0	1	2	1	0	1	2	5,8	51,655	119830	0	155980	905410	0	15,65
Q5SW19	Q5SW19	Clustered mitochondria protein homolog	Cluh	4	0	6	7	4	0	6	7	8	148,07	5846200	0	1,11E+07	9906900	0	88,12
Q3U5Q7	Q3U5Q7	UMP-CMP kinase 2, mitochondrial	Cmpk2	2	0	1	4	2	0	1	4	11,6	50,036	536000	0	772850	1502700	0	23,527
Q6ZQ08	Q6ZQ08	CCR4-NOT transcription complex subunit 1	Cnot1	1	0	1	2	1	0	1	2	1,3	266,8	234290	0	1310500	1670500	0	18,328
Q9DBL7	Q9DBL7	Bifunctional coenzyme A synthase;Phosphopantetheine adenylyltransferase;Dephospho-CoA kinase	Coasy	0	0	1	1	0	0	1	1	2,8	62,022	0	0	293640	146650	0,0017652	6,5048
Q8CIE6	Q8CIE6	Coatomer subunit alpha;Xenin;Proxenin	Copa	1	0	11	20	1	0	11	20	22,2	138,43	791650	0	3,62E+07	9,98E+07	0	212,87
Q9JIF7	Q9JIF7	Coatomer subunit beta	Copb1	1	0	1	9	1	0	1	9	14	107,06	868720	0	3725500	1,82E+07	0	161,28
O55029	O55029	Coatomer subunit beta	Copb2	4	0	4	12	4	0	4	12	16,5	102,45	4063400	0	2,50E+07	2,36E+07	0	81,776
Q9QZE5	Q9QZE5	Coatomer subunit gamma-1	Copg1	5	0	6	10	4	0	5	8	16,5	97,512	1863400	0	1,78E+07	1,30E+07	0	227,83
O88543	O88543	COP9 signalosome complex subunit 3	Cops3	1	0	1	1	1	0	1	1	2,8	47,832	0	0	1015800	151160	0,0010493	8,4697
O89053	O89053	Coronin-1A	Coro1a	5	1	5	10	5	1	5	10	29,1	50,989	6857100	18849	9670500	2,60E+07	0	102,18
Q9WUM4	Q9WUM4	Coronin-1C	Coro1c	0	0	1	1	0	0	1	1	5,7	53,12	0	0	520600	1863600	0	11,639
Q9D2V7	Q9D2V7	Coronin-7	Coro7	1	0	1	3	1	0	1	3	7,3	100,81	348880	0	578280	2866300	0	25,096
P47199	P47199	Quinone oxidoreductase	Cryz	0	0	1	1	0	0	1	1	9,4	35,268	0	0	961700	453860	0	12,86
Q9ERK4	Q9ERK4	Exportin-2	Cse1l	5	3	7	11	5	3	7	11	17,5	110,45	7,05E+07	5242300	5,64E+07	1,44E+07	0	323,31
P70698	P70698	CTP synthase 1	Ctps1	1	1	2	6	1	1	2	6	15,2	66,682	455820	63272	8761500	8934300	0	80,616
Q9WUU7	Q9WUU7	Cathepsin Z	Ctsz	1	1	1	2	1	1	1	2	7,2	33,996	394590	194590	2757000	348800	0	12,041
Q3U308	Q3U308	Cytoplasmic tRNA 2-thiolation protein 2	Ctu2	1	1	1	1	1	1	1	1	3,1	56,104	291180	63471	976640	232950	0,00090992	6,6568
Q9D5V5	Q9D5V5	Cullin-5	Cul5	0	0	1	3	0	0	1	3	6,5	90,973	0	0	601290	2231300	0	26,954
Q7TMB8	Q7TMB8	Cytoplasmic FMR1-interacting protein 1	Cyfip1	1	0	3	11	0	0	1	7	11,1	145,24	3029700	0	5572200	2,19E+07	0	82,545
Q922B2	Q922B2	Aspartate--tRNA ligase, cytoplasmic	Dars	5	2	10	15	5	2	10	15	41,5	57,147	1,45E+07	479910	1,37E+08	6,40E+07	0	323,31
O08788	O08788	Dynactin subunit 1	Dctn1	1	0	1	5	1	0	1	5	5,5	141,67	91302	0	296400	3935300	0	58,785
Q91VR5	Q91VR5	ATP-dependent RNA helicase DDX1	Ddx1	3	0	3	7	3	0	3	7	12,8	82,499	2490700	0	2,04E+07	7531400	0	89,176
Q501J6	Q501J6	Probable ATP-dependent RNA helicase DDX17	Ddx17	4	1	8	10	1	0	3	6	23,5	72,399	112500	0	7766100	2,25E+07	0	245,44
Q61655	Q61655	ATP-dependent RNA helicase DDX19A	Ddx19a	1	0	1	1	1	0	1	1	3,1	53,932	1245600	0	4696100	766570	0	130,12
Q9JIK5	Q9JIK5	Nucleolar RNA helicase 2	Ddx21	3	0	6	16	3	0	6	16	26,6	93,55	2128800	0	6,71E+07	7,64E+07	0	246,7
Q921N6	Q921N6	Probable ATP-dependent RNA helicase DDX27	Ddx27	1	0	3	2	1	0	3	2	6,1	85,938	48691	0	9798600	1336700	0	55,384
62167;P16381;Q62095;Q614	Q62167;P16381;Q62095	ATP-dependent RNA helicase DDX3X;Putative ATP-dependent RNA helicase Pl10;ATP-dependent RNA helicase DDX3Y	Ddx3x;D1Pas1;Ddx3y	8	2	8	10	7	2	7	9	23	73,101	4745300	152860	9,05E+07	2,05E+07	0	189,44
Q569Z5	Q569Z5	Probable ATP-dependent RNA helicase DDX46	Ddx46	0	0	2	2	0	0	2	2	3,7	117,45	0	0	1536900	1221600	0	19,325
Q9CWX9	Q9CWX9	Probable ATP-dependent RNA helicase DDX47	Ddx47	2	1	2	3	2	1	2	3	9,9	50,638	374990	41016	5550800	4016100	0	32,692
Q61656	Q61656	Probable ATP-dependent RNA helicase DDX5	Ddx5	7	1	11	15	4	0	6	11	31,1	69,289	1,93E+07	604640	2,11E+08	7,00E+07	0	312,89
Q9D0R4	Q9D0R4	Probable ATP-dependent RNA helicase DDX56	Ddx56	1	0	2	2	1	0	2	2	9,3	61,211	314180	0	4115200	1581600	0	24,151
P54823	P54823	Probable ATP-dependent RNA helicase DDX6	Ddx6	1	0	1	2	1	0	1	2	8,3	54,191	572850	0	814070	923320	0	16,932
Q7TNV0	Q7TNV0	Protein DEK	Dek	1	0	1	2	1	0	1	2	9,2	43,158	367530	0	4325300	2549300	0	19,991
O35286	O35286	Pre-mRNA-splicing factor ATP-dependent RNA helicase DHX15	Dhx15	7	1	11	15	7	1	11	14	29,3	91,006	9410600	115490	8,72E+07	9,94E+07	0	323,31
Q6PGC1	Q6PGC1	ATP-dependent RNA helicase Dhx29	Dhx29	1	0	1	3	1	0	1	3	2,6	153,97	165800	0	705890	3114900	0	19,67
Q99PU8	Q99PU8	Putative ATP-dependent RNA helicase DHX30	Dhx30	0	0	1	1	0	0	1	1	1,5	136,67	0	0	800670	945330	0	11,358
Q8VHK9;Q6P5D3	Q8VHK9	ATP-dependent RNA helicase DHX36	Dhx36	2	0	3	1	2	0	3	1	4,9	113,88	568000	0	3930800	475630	0	23,533
O70133	O70133	ATP-dependent RNA helicase A	Dhx9	9	2	11	20	9	2	11	20	17,7	149,47	1,31E+07	796610	1,08E+08	1,00E+08	0	323,31
Q3UH60;Q8BWT5	Q3UH60	Disco-interacting protein 2 homolog B	Dip2b	1	0	2	11	1	0	2	11	8,1	171,12	40709	0	2366200	3,11E+07	0	100,45
Q9CSH3	Q9CSH3	Exosome complex exonuclease RRP44	Dis3	3	0	3	4	3	0	3	4	8,1	108,84	1810000	0	3899000	4013300	0	65,952
Q9ESX5	Q9ESX5	H/ACA ribonucleoprotein complex subunit 4	Dkc1	1	1	1	4	1	1	1	4	11,8	57,401	2374700	54714	4951100	6775800	0	31,224
Q9QYJ3	Q9QYJ3	DnaJ homolog subfamily B member 1	Dnajb1	1	0	2	3	1	0	2	3	15,9	38,167	173700	0	1676400	1702700	0	25,85
E9Q8D0	E9Q8D0		Dnajc21	0	0	1	1	0	0	1	1	5,3	61,734	0	0	2182000	936470	0	27,365
Q9QYI3	Q9QYI3	DnaJ homolog subfamily C member 7	Dnajc7	0	0	1	5	0	0	1	5	13,8	56,475	0	0	803460	7758900	0	36,83
Q8K1M6	Q8K1M6	Dynamin-1-like protein	Dnm1l	1	0	1	3	1	0	1	3	6,9	82,657	1001600	0	2440500	1985100	0	54,69
P39054;P39053	P39054	Dynamin-2	Dnm2	2	0	2	1	2	0	2	1	4,3	98,144	2636000	0	3962000	291010	0	23,007
P13864	P13864	DNA (cytosine-5)-methyltransferase 1	Dnmt1	0	0	5	8	0	0	5	8	5,9	183,19	0	0	8932000	9377400	0	51,98

Q8BZN6	Q8BZN6	Dedicator of cytokinesis protein 10	Dock10	2	0	3	8	2	0	3	8	5,6	245,76	643810	0	1039600	8657400	0	97,229
Q8C3J5	Q8C3J5	Dedicator of cytokinesis protein 2	Dock2	0	0	3	11	0	0	3	11	7,3	211,7	0	0	4627300	1,39E+07	0	76,735
P32233	P32233	Developmentally-regulated GTP-binding protein 1	Drg1	1	0	1	4	1	0	1	4	17,4	40,512	144030	0	806290	1569300	0	102,74
Q9QXB9	Q9QXB9	Developmentally-regulated GTP-binding protein 2	Drg2	0	0	1	2	0	0	1	2	11,5	40,718	0	0	272920	457370	0	21,031
O35075	O35075	Down syndrome critical region protein 3 homolog	Dscr3	0	0	1	2	0	0	1	2	12,5	32,97	0	0	537940	239930	0	16,931
Q61495;Q7TSF1	Q61495;Q7TSF1	Desmoglein-1-alpha;Desmoglein-1-beta	Dsg1a;Dsg1b	2	0	2	2	2	0	2	2	1,5	114,6	1771800	0	3083500	870310	0	21,247
E9Q557	E9Q557	Desmoplakin	Dsp	11	1	15	7	11	1	15	7	7,1	332,91	1,18E+07	371490	9,59E+07	6680200	0	259,06
Q9JHU4	Q9JHU4	Cytoplasmic dynein 1 heavy chain 1	Dync1h1	10	2	18	71	10	2	18	71	17,7	532,04	2,24E+07	311250	5,32E+07	2,67E+08	0	323,31
O88487	O88487	Cytoplasmic dynein 1 intermediate chain 2	Dync1i2	0	0	1	2	0	0	1	2	5,7	68,393	0	0	61976	448060	0	13,156
Q8R1Q8	Q8R1Q8	Cytoplasmic dynein 1 light intermediate chain 1	Dync1li1	2	1	1	2	2	1	1	2	6,9	56,614	370690	23383	1309700	5150000	0	39,215
Q9D903	Q9D903	Probable rRNA-processing protein EBP2	Ebna1bp2	0	0	1	1	0	0	1	1	3,9	34,702	0	0	1180100	184070	0,00093897	6,9872
Q6PDI5	Q6PDI5	Proteasome-associated protein ECM29 homolog	Ecm29	0	0	1	2	0	0	1	2	2	203,7	0	0	409360	1176200	0	17,544
P10126;P62631	P10126	Elongation factor 1-alpha 1	Eef1a1	7	1	9	20	7	1	9	20	45,2	50,113	5,97E+07	1988000	2,45E+08	1,49E+09	0	323,31
P57776	P57776	Elongation factor 1-delta	Eef1d	4	1	6	6	4	1	6	6	29,9	31,293	2,82E+07	136330	5,73E+07	2,71E+07	0	323,31
Q9D8N0	Q9D8N0	Elongation factor 1-gamma	Eef1g	6	2	7	12	6	2	7	12	30,4	50,06	3,87E+07	980350	1,12E+08	1,57E+08	0	228,8
P58252	P58252	Elongation factor 2	Eef2	8	1	14	26	7	0	13	25	34,7	95,313	4,74E+07	359210	1,17E+08	2,19E+08	0	323,31
O08810	O08810	116 kDa U5 small nuclear ribonucleoprotein component	Eftud2	1	1	2	3	0	0	1	2	4,8	109,36	0	0	1611500	2995000	0	31,909
Q9WVK4	Q9WVK4	EH domain-containing protein 1	Ehd1	4	0	6	7	2	0	4	5	17,4	60,602	1571400	0	1,58E+07	1,18E+07	0	221,88
Q9EQP2	Q9EQP2	EH domain-containing protein 4	Ehd4	2	1	4	13	2	1	4	12	30,9	61,48	810230	48117	9827100	4,12E+07	0	209,37
Q8BJW6	Q8BJW6	Eukaryotic translation initiation factor 2A;Eukaryotic translation initiation factor 2A, N-terminally processed	Eif2a	1	1	1	2	1	1	1	2	4,8	64,403	1968100	175660	8134100	2089800	0	19,986
Q6ZWX6	Q6ZWX6	Eukaryotic translation initiation factor 2 subunit 1	Eif2s1	6	2	4	10	6	2	4	10	37,5	36,108	1,14E+07	224440	2,06E+07	2,67E+07	0	139,08
Q99L45	Q99L45	Eukaryotic translation initiation factor 2 subunit 2	Eif2s2	2	0	2	7	2	0	2	7	27,2	38,092	1009900	0	2,95E+07	1,02E+07	0	102,17
Q9Z0N1;Q9Z0N2	Q9Z0N1;Q9Z0N2	Eukaryotic translation initiation factor 2 subunit 3, X-linked;Eukaryotic translation initiation factor 2 subunit 3, Y-linked	Eif2s3x;Eif2s3y	3	1	2	12	3	1	2	12	34,1	51,065	1,03E+07	1525800	4,47E+07	9,07E+07	0	168,64
P23116	P23116	Eukaryotic translation initiation factor 3 subunit A	Eif3a	11	0	14	20	11	0	14	20	20,8	161,93	1,20E+07	0	5,29E+07	9,82E+07	0	178,17
Q8JZQ9	Q8JZQ9	Eukaryotic translation initiation factor 3 subunit B	Eif3b	4	0	7	12	4	0	7	12	24,2	91,369	7307200	0	2,64E+07	6,96E+07	0	102,3
Q8R1B4	Q8R1B4	Eukaryotic translation initiation factor 3 subunit C	Eif3c	1	1	4	12	1	1	4	12	18,4	105,53	164300	60440	1,04E+07	3,47E+07	0	323,31
O70194	O70194	Eukaryotic translation initiation factor 3 subunit D	Eif3d	2	1	3	8	2	1	3	8	17,9	63,988	3969600	185030	1,89E+07	2,49E+07	0	69,721
P60229	P60229	Eukaryotic translation initiation factor 3 subunit E	Eif3e	1	0	1	8	1	0	1	8	21,6	52,22	371660	0	6786200	2,23E+07	0	75,908
Q9DCH4	Q9DCH4	Eukaryotic translation initiation factor 3 subunit F	Eif3f	2	1	5	5	2	1	5	5	23,5	37,984	3037500	225960	2,66E+07	1,39E+07	0	120,97
Q91WK2	Q91WK2	Eukaryotic translation initiation factor 3 subunit H	Eif3h	0	0	1	2	0	0	1	2	9,1	39,832	0	0	242500	6131100	0	42,075
Q9QZD9	Q9QZD9	Eukaryotic translation initiation factor 3 subunit I	Eif3i	1	0	1	1	1	0	1	1	2,8	36,46	1854000	0	2477500	1598900	0,00090416	6,6291
Q8QZY1	Q8QZY1	Eukaryotic translation initiation factor 3 subunit L	Eif3l	2	0	4	5	2	0	4	5	16,5	66,612	887680	0	1,67E+07	1,01E+07	0	203,09
Q99JX4	Q99JX4	Eukaryotic translation initiation factor 3 subunit M	Eif3m	0	0	2	2	0	0	2	2	12	42,516	0	0	1668300	913820	0	27,49
P60843;P10630	P60843	Eukaryotic initiation factor 4A-I	Eif4a1	8	3	10	16	7	3	9	15	52,5	46,153	1,04E+08	600960	1,97E+08	1,67E+08	0	323,31
Q91VC3	Q91VC3	Eukaryotic initiation factor 4A-III;Eukaryotic initiation factor 4A-III, N-terminally processed	Eif4a3	4	0	4	6	3	0	3	5	16,5	46,839	2524500	0	4963800	1,52E+07	0	72,363
Q6NZJ6;Q80XI3	Q6NZJ6	Eukaryotic translation initiation factor 4 gamma 1	Eif4g1	0	0	3	8	0	0	3	8	8,6	176,07	0	0	6601800	2,90E+07	0	59,302
Q62448	Q62448	Eukaryotic translation initiation factor 4 gamma 2	Eif4g2	1	1	1	9	1	1	1	9	13,5	102,1	89330	0	1684100	1,42E+07	0	202,45
P70372	P70372	ELAV-like protein 1	Elavl1	1	1	2	2	1	1	2	2	12,9	36,169	1993100	100600	9136300	3916700	0	61,192
Q8BPU7	Q8BPU7	Engulfment and cell motility protein 1	Elmo1	1	1	1	6	1	1	1	6	9,6	83,935	1512500	53057	3104500	6114900	0	44,541
O35130	O35130	Ribosomal RNA small subunit methyltransferase NEP1	Emg1	1	0	1	2	1	0	1	2	8,6	26,974	139230	0	1865700	728910	0	12,537
Q3UMY5	Q3UMY5	Echinoderm microtubule-associated protein-like 4	Eml4	1	0	1	1	1	0	1	1	1,6	110,03	212250	0	4562700	1451600	0,00095602	7,1567
Q61549	Q61549	EGF-like module-containing mucin-like hormone receptor-like 1	Emr1	0	0	1	5	0	0	1	5	5,2	102,13	0	0	1308400	1,16E+07	0	31,055
P17182;P21550;P17183	P17182	Alpha-enolase	Eno1	9	5	11	19	9	5	11	19	54,4	47,14	1,94E+07	1812200	1,61E+08	4,81E+08	0	323,31
Q8CGC7	Q8CGC7	Bifunctional glutamate/proline--tRNA ligase;Glutamate--tRNA ligase;Proline--tRNA ligase	Eprs	6	1	7	30	6	1	7	30	30	170,08	1643200	34565	3,80E+07	1,94E+08	0	323,31
Q9R0P3	Q9R0P3	S-formylglutathione hydrolase	Esd	0	0	3	7	0	0	3	7	34	31,319	0	0	1,01E+07	3,38E+07	0	50,73
Q921I9	Q921I9	Exosome complex component RRP41	Exosc4	1	0	3	1	1	0	3	1	22	26,249	0	0	3555300	148700	0	49,104
Q9CRA8	Q9CRA8	Exosome complex component RRP46	Exosc5	0	0	2	2	0	0	2	2	18,3	25,194	0	0	1120600	194410	0	41,645
Q8BTW3	Q8BTW3	Exosome complex component MTR3	Exosc6	1	1	2	1	1	1	2	1	11,4	28,37	485850	72640	2966600	577050	0	13,951
Q8R1F1	Q8R1F1	Niban-like protein 1	Fam129b	0	0	1	1	0	0	1	1	1,5	84,818	0	0	490780	201490	0,00089445	6,5609
F8VPU2	F8VPU2	FERM, RhoGEF and pleckstrin domain-containing protein 1	Farp1	0	0	1	2	0	0	1	2	2,8	118,87	0	0	1181200	3919800	0	33,999
Q8C0C7	Q8C0C7	Phenylalanine--tRNA ligase alpha subunit	Farsa	3	1	8	9	3	1	8	9	28	57,598	1276200	143450	2,17E+07	1,80E+07	0	277,22
Q9WUA2	Q9WUA2	Phenylalanine--tRNA ligase beta subunit	Farsb	3	1	2	5	3	1	2	5	11,4	65,696	3611300	107050	1,04E+07	6083600	0	61,511
P19096	P19096	Fatty acid synthase;[Acyl-carrier-protein] S-acetyltransferase;[Acyl-carrier-protein] S-malonyltransferase;3-oxoacyl-[acyl-carrier-protein] synthase;3-oxoacyl-[acyl-carrier-protein] reductase;3-hydroxyacyl-[acyl-carrier-protein] dehydratase;Enoyl-[acyl-carrier-protein] reductase;Oleoyl-[acyl-carrier-protein] hydrolase	Fasn	15	0	24	29	15	0	24	29	17	272,43	5,72E+07	0	8,81E+07	1,10E+08	0	323,31
P35550	P35550	rRNA 2-O-methyltransferase fibrillarin	Fbl	2	0	5	8	0	0	3	6	36,1	34,306	1262000	0	2,99E+07	3,59E+07	0	77,969
Q78JE5	Q78JE5	F-box only protein 22	Fbxo22	0	0	1	1	0	0	1	1	3,5	44,202	0	0	870940	210140	0	26,867
P20491	P20491	High affinity immunoglobulin epsilon receptor subunit gamma	Fcer1g	0	0	1	1	0	0	1	1	12,8	9,6523	0	0	211310	955990	0,0017621	6,4921
Q8K1B8	Q8K1B8	Fermitin family homolog 3	Fermt3	4	1	3	10	4	1	3	10	21,5	75,634	6120800	58496	2,00E+07	2,14E+07	0	101,75
P26883	P26883	Peptidyl-prolyl cis-trans isomerase FKBP1A	Fkbp1a	0	0	2	1	0	0	2	1	26,9	11,922	0	0	9807200	194170	0	11,511
P30416	P30416	Peptidyl-prolyl cis-trans isomerase FKBP4;Peptidyl-prolyl cis-trans isomerase FKBP4, N-terminally processed	Fkbp4	1	0	2	5	1	0	2	5	18,1	51,572	875750	0	3352600	6716200	0	39,2
Q9JJ28	Q9JJ28	Protein flightless-1 homolog	Flii	2	0	2	3	2	0	2	3	3,5	144,8	1547100	0	2963300	4101400	0	25,503
Q8BTM8;Q80X90;Q8VHX6	Q8BTM8	Filamin-A	Flna	3	0	6	12	3	0	6	12	7,7	281,22	2091300	0	4098000	3,16E+07	0	205,26
P10404;P11370	P10404;P11370	MLV-related proviral Env polyprotein;Surface protein;Transmembrane protein;Retrovirus-related Env polyprotein from Fv-4 locus	Fv4	1	0	1	3	1	0	1	3	5	69,612	101050	0	1339300	5590200	0	39,26
P97855	P97855	Ras GTPase-activating protein-binding protein 1	G3bp1	1	0	3	2	1	0	3	2	9	51,828	165660	0	3002300	761500	0	33,324
00612;P97324;REV__P4666	Q00612	Glucose-6-phosphate 1-dehydrogenase X	G6pdx	3	0	4	12	3	0	4	12	27,8	59,262	3499400	0	1,54E+07	3,36E+07	0	323,31
Q9R0N0	Q9R0N0	Galactokinase	Galk1	2	1	5	2	2	1	5	2	16,3	42,295	658520	37298	1,05E+07	1655000	0	45,665
P16858;Q64467	P16858	Glyceraldehyde-3-phosphate dehydrogenase	Gapdh	7	2	10	17	7	2	10	17	53,8	35,81	1,42E+08	8860300	7,87E+08	8,12E+08	0	323,31
Q9CZD3	Q9CZD3	Glycine--tRNA ligase	Gars	1	0	4	10	1	0	4	10	19,8	81,877	4753900	0	1,87E+07	3,23E+07	0	96,803

Q64737	Q64737	Trifunctional purine biosynthetic protein adenosine-3;Phosphoribosylamine--glycine ligase;Phosphoribosylformylglycinamidine cyclo-ligase;Phosphoribosylglycinamide formyltransferase	Gart	2	0	2	5	2	0	2	5	8	107,5	6375800	0	8166200	1,24E+07	0	49,972
Q8VHR5	Q8VHR5	Transcriptional repressor p66-beta	Gatad2b	1	0	1	3	1	0	1	3	8,2	65,41	659650	0	2057700	1650300	0	21,565
E9PVA8	E9PVA8		Gcn1l1	2	0	7	25	2	0	7	25	13	293,02	507910	0	5746800	4,11E+07	0	323,31
Q61598;P50396	Q61598;P50396	Rab GDP dissociation inhibitor beta;Rab GDP dissociation inhibitor alpha	Gdi2;Gdi1	1	0	1	2	1	0	1	2	5,6	50,537	454410	0	1183600	7063200	0	158,18
P47856;Q9Z2Z9	P47856	Glutamine--fructose-6-phosphate aminotransferase [isomerizing] 1	Gfpt1	0	0	1	9	0	0	1	9	17,2	78,538	0	0	793440	1,26E+07	0	190,85
Q8BTZ7	Q8BTZ7	Mannose-1-phosphate guanyltransferase beta	Gmppb	2	0	2	1	2	0	2	1	10,6	39,916	463080	0	4395300	545020	0	21,432
Q99L27	Q99L27	GMP reductase 2	Gmpr2	1	1	1	2	1	1	1	2	8,6	38,018	459990	95078	963050	1221400	0	12,784
P27601	P27601	Guanine nucleotide-binding protein subunit alpha-13	Gna13	1	0	1	3	1	0	1	3	9	44,054	195450	0	1118400	1829200	0	52,875
P08752;B2RSH2	P08752	Guanine nucleotide-binding protein G(i) subunit alpha-2	Gnai2	1	0	2	10	1	0	2	8	33	40,489	1718000	0	5297900	3,87E+07	0	80,398
18872;P20612;Q3V3I2;P5014	Q9DC51	Guanine nucleotide-binding protein G(k) subunit alpha	Gnai3	1	0	1	6	1	0	1	4	21,8	40,538	565340	0	2632400	1,02E+07	0	26,069
P63094;Q6R0H7	P63094;Q6R0H7	Guanine nucleotide-binding protein G(s) subunit alpha isoforms short;Guanine nucleotide-binding protein G(s) subunit alpha isoforms XLas	Gnas	1	1	1	4	1	1	1	3	12,7	45,663	145480	66645	1207000	7491800	0	19,18
P62880;P29387;Q61011	P62880;P29387	Guanine nucleotide-binding protein G(I)/G(S)/G(T) subunit beta-2;Guanine nucleotide-binding protein subunit beta-4	Gnb2;Gnb4	2	0	1	7	2	0	1	4	26,8	37,331	581290	0	3837700	2,62E+07	0	48,704
P68040	P68040	Guanine nucleotide-binding protein subunit beta-2-like 1;Guanine nucleotide-binding protein subunit beta-2-like 1, N-terminally processed	Gnb2l1	8	2	7	16	8	2	7	16	56,5	35,076	2,34E+07	826490	5,12E+07	1,13E+08	0	323,31
P05201	P05201	Aspartate aminotransferase, cytoplasmic	Got1	1	0	1	5	1	0	1	5	17,9	46,247	156160	0	1082000	3109800	0	36,167
Q8BUV3	Q8BUV3	Gephyrin;Molybdopterin adenylyltransferase;Molybdopterin molybdenumtransferase	Gphn	1	1	1	1	1	1	1	1	1,8	83,281	457110	19185	523410	317430	0,0025862	6,417
P10649	P10649	Glutathione S-transferase Mu 1	Gstm1	1	0	1	1	1	0	1	1	5,5	25,97	213110	0	820100	95693	0,00093985	6,991
P19157;P46425	P19157;P46425	Glutathione S-transferase P 1;Glutathione S-transferase P 2	Gstp1;Gstp2	0	0	2	2	0	0	2	2	12,9	23,609	0	0	1,52E+07	870950	0	89,311
)5;P01897;P01896;P01898;P	01899;P01900;P01895;P0189	H-2 class I histocompatibility antigen, D-B alpha chain;H-2 class I histocompatibility antigen, D-D alpha chain;H-2 class I histocompatibility antigen, alpha chain;H-2 class I histocompatibility antigen, L-D alpha chain	H2-D1;H2-L	2	1	2	4	1	0	1	2	14,1	40,836	1484200	0	2054200	5159900	0	12,846
P14439	P14439	H-2 class II histocompatibility antigen, E-U alpha chain	H2-Ea	0	0	1	1	0	0	1	1	3,9	29,107	0	0	359070	1564700	0	11,543
)4223;P14428;P03991;P144	01901;P04223;P14428;P0399	H-2 class I histocompatibility antigen, K-B alpha chain;H-2 class I histocompatibility antigen, K-K alpha chain;H-2 class I histocompatibility antigen, K-Q alpha chain;H-2 class I histocompatibility antigen, K-W28 alpha chain	H2-K1	2	1	2	6	1	0	1	4	27,9	41,301	3236700	52163	7876100	1,44E+07	0	45,294
84244;P84228;P68433;P0230	84244;P84228;P68433;P0230	Histone H3.3;Histone H3.2;Histone H3.1;Histone H3.3C	a;Hist1h3b;Hist1h3a;H	6	4	6	3	6	4	6	3	33,1	15,328	2,04E+08	7858100	5,56E+08	2,58E+07	0	41,673
Q8BY71	Q8BY71	Histone acetyltransferase type B catalytic subunit	Hat1	2	1	4	3	2	1	4	3	17,8	49,278	3667000	89451	1,00E+07	1456100	0	71,68
Q61191	Q61191	Host cell factor 1;HCF N-terminal chain 1;HCF N-terminal chain 2;HCF N-terminal chain 3;HCF N-terminal chain 4;HCF N-terminal chain 5;HCF N-terminal chain 6;HCF C-terminal chain 1;HCF C-terminal chain 2;HCF C-terminal chain 3;HCF C-terminal chain 4;HCF C-terminal chain 5;HCF C-terminal chain 6	Hcfc1	0	0	1	1	0	0	1	1	0,8	210,43	0	0	352470	108230	0,00091075	6,663
Q8VDJ3	Q8VDJ3	Vigilin	Hdlbp	1	0	2	8	1	0	2	8	8,7	141,74	251840	0	1552600	1,49E+07	0	76,458
E9QAM5	E9QAM5	Helicase with zinc finger domain 2	Helz2	0	0	1	6	0	0	1	6	2,6	331,56	0	0	262920	3122200	0	42,305
Q9JLZ6	Q9JLZ6	Hypermethylated in cancer 2 protein	Hic2	1	1	1	1	1	1	1	1	1,6	66,766	4,95E+07	1,40E+07	3,48E+08	6,44E+07	0	61,721
P43276	P43276	Histone H1.5	Hist1h1b	1	1	1	1	1	1	1	1	7,2	22,576	1158400	113760	1582300	337130	0,0010395	8,2528
P15864;Q07133;P43275	P15864	Histone H1.2	Hist1h1c	2	1	3	4	1	1	1	2	20,8	21,266	1190100	299880	4486900	3410500	0	31,226
P43277	P43277	Histone H1.3	Hist1h1d	2	1	3	3	1	1	1	1	18,1	22,099	3275400	569160	1634800	708840	0,0010246	7,8211
HKE2;C0HKE1;Q8CGP6;Q8R	;C0HKE3;C0HKE2;C0HKE1;	Histone H2A type 1-H;Histone H2A.J;Histone H2A type 1-K;Histone H2A type 1-F;Histone H2A type 3;Histone H2AX	;Hist1h2ak;Hist1h2af	4	2	6	3	1	0	2	1	35,4	14,135	9,49E+07	5,65E+07	7,22E+08	937600	0	145,5
Q64478;Q64475;P10854;P10	Q6ZWY9;Q64525;Q64478;Q64	Histone H2B type 1-P;Histone H2B type 1-K;Histone H2B type 1-C/E/G;Histone H2B type 2-B;Histone H2B type 1-H;Histone H2B type 1-B;Histone H2B type 1-M;Histone H2B type 1-F/J/L	Hist2h2bb;Hist1h2bh;	2	1	3	2	2	1	3	2	30,2	13,992	6365800	162160	3,84E+07	6759100	0	76,619
P62806	P62806	Histone H4	Hist1h4a	4	2	5	6	4	2	5	6	50,5	11,367	1,32E+08	7003700	1,58E+08	8,27E+07	0	114,57
Q3TRM8;O08528	Q3TRM8	Hexokinase-3	Hk3	6	2	8	4	6	2	8	4	14	100,1	8762900	556740	2,59E+07	2481000	0	132,36
P49312	P49312	Heterogeneous nuclear ribonucleoprotein A1;Heterogeneous nuclear ribonucleoprotein A1, N-terminally processed	Hnrnpa1	0	0	1	1	0	0	1	1	5	34,196	0	0	3535900	624350	0,0033898	6,3631
O88569	O88569	Heterogeneous nuclear ribonucleoproteins A2/B1	Hnrnpa2b1	1	1	3	4	1	1	3	4	18,1	37,402	315330	420940	3,97E+07	3354000	0	30,49
Q8BG05	Q8BG05	Heterogeneous nuclear ribonucleoprotein A3	Hnrnpa3	2	1	2	3	2	1	2	3	9,2	39,652	283130	80398	8843600	4634700	0	25,171
Q99020	Q99020	Heterogeneous nuclear ribonucleoprotein A/B	Hnrnpab	1	0	2	5	1	0	2	5	18,9	30,831	73156	0	1830000	8553700	0	37,093
Q9Z204	Q9Z204	Heterogeneous nuclear ribonucleoproteins C1/C2	Hnrnpc	2	1	3	6	2	1	3	6	19,8	34,384	506120	15184	1,65E+07	3,71E+07	0	66,91
O35737;P70333	O35737;P70333	Heterogeneous nuclear ribonucleoprotein H;Heterogeneous nuclear ribonucleoprotein H, N-terminally processed;Heterogeneous nuclear ribonucleoprotein H2	Hnrnph1;Hnrnph2	2	0	5	6	1	0	3	5	25,8	49,199	7303000	0	5,54E+07	1,30E+07	0	132,12
P61979	P61979	Heterogeneous nuclear ribonucleoprotein K	Hnrnpk	6	2	7	6	6	2	7	6	18,4	50,976	1,03E+07	842330	3,94E+07	1,70E+07	0	85,061
Q8R081	Q8R081	Heterogeneous nuclear ribonucleoprotein L	Hnrnpl	0	0	1	6	0	0	1	6	19,6	63,963	0	0	355220	1,49E+07	0	55,48
Q9D0E1	Q9D0E1	Heterogeneous nuclear ribonucleoprotein M	Hnrnpm	4	0	5	6	4	0	5	6	15,8	77,648	1238900	0	1,69E+07	9945400	0	77,21
Q8VEK3	Q8VEK3	Heterogeneous nuclear ribonucleoprotein U	Hnrnpu	2	0	5	16	2	0	5	16	21,5	87,917	7312200	0	7,35E+07	2,30E+08	0	323,31
Q00PI9	Q00PI9	Heterogeneous nuclear ribonucleoprotein U-like protein 2	Hnrnpul2	0	0	1	1	0	0	1	1	5,5	84,939	0	0	2553500	539290	0	43,865
Q61411;P08556;P32883	Q61411;P08556;P32883	GTPase HRas;GTPase HRas, N-terminally processed;GTPase NRas;GTPase KRas;GTPase KRas, N-terminally processed	Hras;Nras;Kras	0	0	1	3	0	0	1	3	14,3	21,298	0	0	253680	1063100	0	17,723
P07901	P07901	Heat shock protein HSP 90-alpha	Hsp90aa1	9	4	12	22	5	3	6	13	31	84,787	4,39E+07	280320	1,20E+08	5,61E+07	0	309,35
P11499;P08113	P11499	Heat shock protein HSP 90-beta	Hsp90ab1	8	2	14	22	4	1	8	13	31,6	83,28	1,10E+08	1,26E+07	2,06E+08	2,51E+08	0	323,31
Q61316	Q61316	Heat shock 70 kDa protein 4	Hspa4	3	1	3	9	2	1	3	8	15,1	94,132	6853000	104210	6153800	2,51E+07	0	221,05
P20029	P20029	78 kDa glucose-regulated protein	Hspa5	3	1	4	6	1	0	2	4	14,4	72,421	523200	0	5144300	4681900	0	104,59
P63017;P17156	P63017	Heat shock cognate 71 kDa protein	Hspa8	16	7	18	26	13	5	15	22	44,7	70,87	1,47E+08	1,27E+07	5,37E+08	5,91E+08	0	323,31
P63038	P63038	60 kDa heat shock protein, mitochondrial	Hspd1	1	1	1	2	1	1	1	2	9,4	60,955	170880	52340	2370000	2178600	0	24,525
Q61699;P48722	Q61699	Heat shock protein 105 kDa	Hsph1	1	0	1	5	0	0	1	4	7,5	96,406	0	0	3052200	2686300	0	35,551
Q8BU30	Q8BU30	Isoleucine--tRNA ligase, cytoplasmic	Iars	4	1	7	18	4	1	7	18	16,2	144,27	1,55E+07	70869	2,44E+07	7,02E+07	0	170,32

Q9JHR7	Q9JHR7	Insulin-degrading enzyme	Ide	0	0	1	1	0	0	1	1	2	117,77	0	0	777590	1155800	0	11,74
Q8R366	Q8R366	Immunoglobulin superfamily member 8	Igsf8	2	2	4	3	2	2	4	3	10	65,01	777900	291290	1,67E+07	9749200	0	139,86
Q7TT37	Q7TT37	Elongator complex protein 1	Ikbkap	1	0	2	7	1	0	2	7	6,5	149,58	144230	0	3371000	7380100	0	67,171
Q921Y2	Q921Y2	U3 small nucleolar ribonucleoprotein protein IMP3	Imp3	1	0	1	1	1	0	1	1	8,2	21,777	119020	0	3011000	318020	0,00097087	7,2762
P24547	P24547	Inosine-5-monophosphate dehydrogenase 2	Impdh2	1	0	2	14	1	0	2	14	32,3	55,814	469850	0	2973600	3,55E+07	0	162,83
Q9ES52	Q9ES52	Phosphatidylinositol 3,4,5-trisphosphate 5-phosphatase 1	Inpp5d	0	0	1	1	0	0	1	1	0,8	133,54	0	0	2279200	694220	0,0033585	6,3332
Q7TQK1	Q7TQK1	Integrator complex subunit 7	Ints7	1	1	1	1	1	1	1	1	1,4	106,86	3248600	149200	1913600	8082600	0,0025929	6,4263
Q8BKC5	Q8BKC5	Importin-5	Ipo5	1	0	1	12	1	0	1	12	15,9	123,59	520740	0	1011800	1,85E+07	0	237,95
Q9EPL8	Q9EPL8	Importin-7	Ipo7	0	0	2	2	0	0	2	2	5,2	119,49	0	0	612890	1968200	0	24,699
Q9JKF1	Q9JKF1	Ras GTPase-activating-like protein IQGAP1	Iqgap1	1	1	5	34	1	1	5	32	24,5	188,74	769520	76412	3467900	1,23E+08	0	323,31
P54987	P54987	Cis-aconitate decarboxylase	Irg1	1	1	2	2	1	1	2	2	10,9	53,758	448800	173210	3,21E+07	2347300	0	25,137
Q00651	Q00651	Integrin alpha-4	Itga4	0	0	2	4	0	0	2	4	5,4	115,69	0	0	1183200	1,14E+07	0	30,396
P11688	P11688	Integrin alpha-5;Integrin alpha-5 heavy chain;Integrin alpha-5 light chain	Itga5	1	0	1	1	1	0	1	1	2,1	115,04	297700	0	1054900	781560	0	14,244
P09055	P09055	Integrin beta-1	Itgb1	0	0	1	1	0	0	1	1	3,5	88,231	0	0	662140	1459000	0	12,306
P11835	P11835	Integrin beta-2	Itgb2	1	1	1	3	1	1	1	3	7,3	85,025	263960	139290	2280700	4390300	0	83,152
Q02257	Q02257	Junction plakoglobin	Jup	6	2	12	7	6	2	12	7	24,7	81,8	7142400	298750	8,40E+07	2,01E+07	0	323,31
Q99MN1	Q99MN1	Lysine--tRNA ligase	Kars	0	0	2	4	0	0	2	4	12,3	67,839	0	0	1308800	6681800	0	163,23
Q3U0V1	Q3U0V1	Far upstream element-binding protein 2	Khsrp	2	2	2	1	2	2	2	1	2,8	76,775	4012100	385300	2479600	299580	0	56,918
Q8BKS9	Q8BKS9	Pumilio domain-containing protein KIAA0020	Kiaa0020	0	0	1	1	0	0	1	1	3,6	72,799	0	0	1878500	465930	0	12,998
Q8C2E7	Q8C2E7	WASH complex subunit strumpellin	Kiaa0196	0	0	1	1	0	0	1	1	1,2	134,11	0	0	310880	531080	0,0010604	9,899
Q5DTW7	Q5DTW7	Uncharacterized protein KIAA1551	Kiaa1551	0	0	1	1	0	0	1	1	2,4	169,04	0	0	345970	1064700	0	11,98
Q61768;P28738;P33175	Q61768;P28738;P33175	Kinesin-1 heavy chain;Kinesin heavy chain isoform 5C;Kinesin heavy chain isoform 5A	Kif5b;Kif5c;Kif5a	2	0	2	6	2	0	2	6	7,7	109,55	867270	0	2166200	1,02E+07	0	56,108
P52293	P52293	Importin subunit alpha-1	Kpna2	4	0	3	4	4	0	3	4	17,4	57,927	1300400	0	5581100	4709200	0	307,96
P70168	P70168	Importin subunit beta-1	Kpnb1	3	0	5	7	3	0	5	7	12,8	97,183	715400	0	9926900	8732400	0	182,35
Q3UV17	Q3UV17	Keratin, type II cytoskeletal 2 oral	Krt76	6	5	9	9	1	1	1	2	13,1	62,844	7,09E+07	112600	2689300	5791800	0	12,926
Q6ZQ58	Q6ZQ58	La-related protein 1	Larp1	2	0	1	2	2	0	1	2	2,9	121,12	629770	0	1801000	3252000	0	13,54
Q8BMJ2	Q8BMJ2	Leucine--tRNA ligase, cytoplasmic	Lars	4	0	5	11	4	0	5	11	13,8	134,19	4213900	0	1,14E+07	4,79E+07	0	149,24
Q61233;Q99K51	Q61233	Plastin-2	Lcp1	2	1	1	5	2	1	1	5	11,5	70,148	1879600	273690	803710	6434400	0	36,51
P06151;P00342;P16125	P06151	L-lactate dehydrogenase A chain	Ldha	1	1	1	9	1	1	1	9	28,3	36,498	1189200	34728	9409100	2,10E+07	0	60,89
P16110	P16110	Galectin-3	Lgals3	0	0	1	3	0	0	1	3	11	27,515	0	0	2195900	4608400	0	27,728
P37913	P37913	DNA ligase 1	Lig1	1	0	1	7	1	0	1	7	9,4	102,29	1113600	0	17491	1,17E+07	0	41,357
P48678	P48678	Prelamin-A/C;Lamin-A/C	Lmna	1	0	1	1	1	0	1	1	2,4	74,237	288000	0	4716700	1701400	0,00099206	7,4519
P11152	P11152	Lipoprotein lipase	Lpl	1	0	2	8	1	0	2	8	19,6	53,109	185530	0	5187300	8,36E+07	0	156,28
Q8K1T1	Q8K1T1	Leucine-rich repeat-containing protein 25	Lrrc25	1	0	1	5	1	0	1	5	27,3	32,673	791150	0	2396600	4,24E+07	0	97,174
P25911	P25911	Tyrosine-protein kinase Lyn	Lyn	1	1	1	3	1	1	1	3	9,8	58,812	147600	39505	751130	1971400	0	25,325
P17897	P17897	Lysozyme C-1	Lyz1	1	1	1	1	1	1	1	1	8,1	16,794	1794600	123260	3325800	557990	0	46,322
P24668	P24668	Cation-dependent mannose-6-phosphate receptor	M6pr	1	0	1	1	1	0	1	1	5	31,172	2512300	0	1155600	4596300	0	19,202
Q8C052	Q8C052	Microtubule-associated protein 1S;MAP1S heavy chain;MAP1S light chain	Map1s	2	0	3	1	2	0	3	1	4	102,94	2825600	0	9174400	1322100	0	19,734
P63085	P63085	Mitogen-activated protein kinase 1	Mapk1	2	0	2	2	2	0	2	2	7,8	41,275	5428200	0	1,09E+07	1233600	0	22,591
Q63844	Q63844	Mitogen-activated protein kinase 3	Mapk3	1	1	1	1	1	1	1	1	3,9	43,066	1543100	137330	2386400	160040	0,00097943	7,3079
Q61166	Q61166	Microtubule-associated protein RP/EB family member 1	Mapre1	1	0	2	3	1	0	2	3	20,5	30,016	286750	0	1874100	4372800	0	90,805
Q68FL6	Q68FL6	Methionine--tRNA ligase, cytoplasmic	Mars	3	1	6	15	3	1	6	15	24,8	101,43	1236800	25940	1,90E+07	7,30E+07	0	186,18
Q3THS6	Q3THS6	S-adenosylmethionine synthase isoform type-2	Mat2a	2	1	2	4	2	1	2	4	13,7	43,688	4384300	139530	1,68E+07	1,75E+07	0	57,425
Q99LB6	Q99LB6	Methionine adenosyltransferase 2 subunit beta	Mat2b	1	1	1	3	1	1	1	3	15	37,392	3589900	337880	4861400	3197800	0	66,44
P97310	P97310	DNA replication licensing factor MCM2	Mcm2	7	1	10	11	7	1	10	11	21,1	102,08	6111800	44064	3,20E+07	2,07E+07	0	137,59
P25206	P25206	DNA replication licensing factor MCM3	Mcm3	8	1	11	9	8	1	11	9	22,5	91,545	1,28E+07	89247	7,24E+07	8213000	0	206,86
P49717	P49717	DNA replication licensing factor MCM4	Mcm4	3	0	8	16	3	0	8	16	28,2	96,735	1760600	0	1,33E+07	4,14E+07	0	323,31
P49718	P49718	DNA replication licensing factor MCM5	Mcm5	3	0	4	7	3	0	4	7	16,2	82,342	7615400	0	3,70E+07	1,35E+07	0	83,966
P97311	P97311	DNA replication licensing factor MCM6	Mcm6	5	0	6	2	5	0	6	2	9,6	92,866	1,18E+07	0	3,82E+07	465880	0	97,064
Q61881	Q61881	DNA replication licensing factor MCM7	Mcm7	8	1	9	10	8	1	9	10	25,3	81,21	7869200	42889	5,03E+07	2,80E+07	0	260,94
Q8R3C0	Q8R3C0	Mini-chromosome maintenance complex-binding protein	Mcmbp	1	0	1	1	1	0	1	1	2	72,89	488130	0	1302500	144720	0,0034276	6,395
P08249	P08249	Malate dehydrogenase, mitochondrial	Mdh2	1	0	1	6	1	0	1	6	28,7	35,611	130670	0	130100	2163300	0	48,064
Q91VH6	Q91VH6	Protein MEMO1	Memo1	1	0	4	1	1	0	4	1	17,2	33,692	686940	0	2,07E+07	495450	0	55,624
Q8BP48	Q8BP48	Methionine aminopeptidase 1	Metap1	0	0	1	1	0	0	1	1	5,7	43,221	0	0	638240	469610	0	12,643
P34884	P34884	Macrophage migration inhibitory factor	Mif	1	1	1	2	1	1	1	2	17,4	12,504	2,08E+07	122520	1,11E+07	1958800	0	76,324
A2A7Y5	A2A7Y5	Migration and invasion-inhibitory protein	Miip	1	1	1	1	1	1	1	1	3,6	42,828	915250	294710	1,33E+07	1,33E+07	0,00815	6,2231
Q8CD15	Q8CD15	Bifunctional lysine-specific demethylase and histidyl-hydroxylase MINA	Mina	1	1	3	3	1	1	3	3	7,3	53,516	996440	69578	3854800	2721800	0	24,731
P23249	P23249	Putative helicase MOV-10	Mov10	1	0	1	11	1	0	1	11	15,8	113,58	297610	0	441490	2,49E+07	0	138,42
Q9CQT1	Q9CQT1	Methylthioribose-1-phosphate isomerase	Mri1	3	1	3	2	3	1	3	2	17,3	39,41	2426500	159660	2337200	1151900	0	38,202
P26041	P26041	Moesin	Msn	0	0	2	21	0	0	1	18	35,7	67,766	0	0	1992200	1,18E+08	0	323,31
Q2YDW2	Q2YDW2	Protein misato homolog 1	Msto1	2	1	4	5	2	1	4	5	13,3	61,23	1242200	165740	9344200	3774900	0	45,187
Q9R190;Q924K8;Q8K4B0	Q9R190	Metastasis-associated protein MTA2	Mta2	1	0	4	7	1	0	4	7	19,9	75,029	288600	0	5913300	9932600	0	103,33
Q9CQ65	Q9CQ65	S-methyl-5-thioadenosine phosphorylase	Mtap	0	0	1	1	0	0	1	1	10,2	31,062	0	0	666160	1236700	0	11,529
Q922D8	Q922D8	C-1-tetrahydrofolate synthase, cytoplasmic;Methylenetetrahydrofolate dehydrogenase;Methenyltetrahydrofolate cyclohydrolase;Formyltetrahydrofolate synthetase;C-1-tetrahydrofolate synthase, cytoplasmic, N-terminally processed	Mthfd1	3	0	6	19	3	0	6	19	28,9	101,2	3758600	0	3,48E+07	8,10E+07	0	205,53
Q9EQK5	Q9EQK5	Major vault protein	Mvp	0	0	2	21	0	0	2	21	31,9	95,923	0	0	543840	1,07E+08	0	323,31
O35682	O35682	Myeloid-associated differentiation marker	Myadm	1	0	1	2	1	0	1	2	7,2	35,284	87250	0	423750	5582500	0	16,035
Q7TPV4	Q7TPV4	Myb-binding protein 1A	Mybbp1a	7	2	11	23	7	2	11	23	22,4	152,04	1,25E+07	207350	9,69E+07	7,52E+07	0	323,31
[illegible]5;P13542;Q5SX39;P13541;[illegible]	Q8VDD5	Myosin-9	Myh9	2	1	19	81	2	1	17	67	41,5	226,37	244160	267240	1,80E+07	7,50E+08	0	323,31
Q9JMH9	Q9JMH9	Unconventional myosin-XVIIIa	Myo18a	1	0	1	5	1	0	1	5	3,1	232,75	202030	0	133620	5012100	0	39,518
Q99104;P21271	Q99104	Unconventional myosin-Va	Myo5a	0	0	1	4	0	0	1	4	3	215,54	0	0	697710	4055100	0	199,9
Q3UX61;Q9QY36	Q3UX61;Q9QY36	N-alpha-acetyltransferase 11;N-alpha-acetyltransferase 10	Naa11;Naa10	1	1	1	1	1	1	1	1	10,6	24,67	2479100	96982	912510	587280	0,001087	11,189
Q80UM3;Q9DBB4	Q80UM3	N-alpha-acetyltransferase 15, NatA auxiliary subunit	Naa15	3	2	3	13	3	2	3	13	16,5	100,96	6867900	477370	3,32E+07	5,84E+07	0	291,04

Q8CES0	Q8CES0	N-alpha-acetyltransferase 30	Naa30	0	0	1	1	0	0	1	1	4,9	39,432	0	0	57192	188280	0,00093371	6,9145
P28656	P28656	Nucleosome assembly protein 1-like 1	Nap1l1	0	0	1	1	0	0	1	1	4,3	45,345	0	0	1499800	325260	0,0010718	10,314
Q8BP47	Q8BP47	Asparagine--tRNA ligase, cytoplasmic	Nars	3	1	5	10	3	1	5	10	24,9	64,279	3786400	10204	3,32E+07	3,66E+07	0	323,31
P97369	P97369	Neutrophil cytosol factor 4	Ncf4	1	0	1	2	1	0	1	2	7,7	38,707	116790	0	632120	1783400	0	16,819
P09405	P09405	Nucleolin	Ncl	2	0	2	11	2	0	2	11	23,2	76,722	392670	0	6,38E+07	5,26E+07	0	129,41
Q8C4Y3	Q8C4Y3	Negative elongation factor B	Nelfb	1	0	1	1	1	0	1	1	1,7	65,636	60188	0	213570	170740	0,00090009	6,5969
Q9WTK5	Q9WTK5	Nuclear factor NF-kappa-B p100 subunit;Nuclear factor NF-kappa-B p52 subunit	Nfkb2	0	0	1	1	0	0	1	1	1,6	96,831	0	0	525610	613890	0,00098039	7,3137
Q9D0T1	Q9D0T1	NHP2-like protein 1;NHP2-like protein 1, N-terminally processed	Nhp2l1	0	0	1	1	0	0	1	1	9,4	14,173	0	0	580730	499160	0,00097371	7,2882
Q80TM9	Q80TM9	Nischarin	Nisch	1	0	1	2	1	0	1	2	1,7	175,01	573460	0	1555500	2019000	0	12,018
Q8VEJ4	Q8VEJ4	Notchless protein homolog 1	Nle1	1	0	1	1	1	0	1	1	3,1	53,133	2622800	0	4496700	722300	0,0010661	10,136
Q01768;P15532	Q01768;P15532	Nucleoside diphosphate kinase B;Nucleoside diphosphate kinase A	Nme2;Nme1	1	0	1	2	1	0	1	2	13,8	17,363	446080	0	2268600	3862700	0	11,561
Q9JJF3	Q9JJF3	Bifunctional lysine-specific demethylase and histidyl-hydroxylase NO66	No66	2	1	2	2	2	1	2	2	5,8	67,556	657530	26189	5249000	849800	0	12,162
Q9WV70	Q9WV70	Nucleolar complex protein 2 homolog	Noc2l	1	0	2	2	1	0	2	2	3,6	85,423	72012	0	4069600	948440	0	26,701
Q8R5K4	Q8R5K4	Nucleolar protein 6	Nol6	0	0	2	1	0	0	2	1	3,9	129,23	0	0	874030	792000	0	19,138
Q99K48	Q99K48	Non-POU domain-containing octamer-binding protein	Nono	2	0	3	5	1	0	2	4	13,5	54,54	5484300	0	1,23E+07	8937200	0	30,17
Q922K7	Q922K7	Probable 28S rRNA (cytosine-C(5))-methyltransferase	Nop2	1	0	3	4	1	0	3	4	7,8	86,751	2791300	0	3,49E+07	1,30E+07	0	49,359
Q9D6Z1	Q9D6Z1	Nucleolar protein 56	Nop56	1	0	6	11	1	0	6	11	25,2	64,464	558910	0	2,59E+07	3,42E+07	0	212,14
Q6DFW4	Q6DFW4	Nucleolar protein 58	Nop58	3	0	5	6	3	0	5	6	18,3	60,342	1943500	0	3,78E+07	1,39E+07	0	112,62
Q8BMC4	Q8BMC4	Nucleolar protein 9	Nop9	0	0	2	2	0	0	2	2	4,6	70,046	0	0	1463900	248060	0	14,436
P97300	P97300	Neuroplastin	Nptn	0	0	1	1	0	0	1	1	4,3	44,373	0	0	842520	761780	0,00089127	6,5391
P46460	P46460	Vesicle-fusing ATPase	Nsf	1	0	1	7	1	0	1	7	13,3	82,613	806920	0	1994700	1,19E+07	0	90,132
Q1HFZ0	Q1HFZ0	tRNA (cytosine(34)-C(5))-methyltransferase	Nsun2	1	0	1	1	1	0	1	1	3	85,451	146820	0	1173000	283800	0	11,957
Q9JKX6	Q9JKX6	ADP-sugar pyrophosphatase	Nudt5	1	0	1	2	1	0	1	2	10,1	23,984	210970	0	714850	1199600	0	12,619
Q80U93	Q80U93	Nuclear pore complex protein Nup214	Nup214	0	0	1	2	0	0	1	2	1,4	212,98	0	0	346630	1554600	0	15,945
Q8VI94	Q8VI94	2-5-oligoadenylate synthase-like protein 1	Oasl1	2	0	3	12	2	0	3	12	30,7	59,088	304570	0	3314000	7,59E+07	0	109,08
Q8CGY8	Q8CGY8	UDP-N-acetylglucosamine--peptide N-acetylglucosaminyltransferase 110 kDa subunit	Ogt	0	0	1	1	0	0	1	1	2,6	116,95	0	0	365760	686920	0	13,697
Q8BWU5	Q8BWU5	Probable tRNA N6-adenosine threonylcarbamoyltransferase	Osgep	1	0	1	3	1	0	1	3	14,6	36,3	390680	0	909890	1179400	0	19,324
P50580	P50580	Proliferation-associated protein 2G4	Pa2g4	3	1	4	10	3	1	4	10	31	43,698	1,11E+07	287050	1,53E+07	4,57E+07	0	161,24
P29341	P29341	Polyadenylate-binding protein 1	Pabpc1	3	2	4	21	3	2	4	21	33,8	70,67	2418500	779190	1,60E+07	1,45E+08	0	323,31
Q9DCE5	Q9DCE5	p21-activated protein kinase-interacting protein 1	Pak1ip1	0	0	2	4	0	0	2	4	16	42,116	0	0	3153800	2981400	0	42,069
P11103	P11103	Poly [ADP-ribose] polymerase 1	Parp1	1	0	1	9	1	0	1	9	11,9	113,1	708540	0	3034800	1,54E+07	0	62,17
P60335	P60335	Poly(rC)-binding protein 1	Pcbp1	3	1	3	4	1	0	1	2	16,9	37,497	280790	0	3199600	1845600	0	11,765
Q61990;P57722	Q61990;P57722	Poly(rC)-binding protein 2;Poly(rC)-binding protein 3	Pcbp2;Pcbp3	4	1	5	5	2	0	3	3	26,2	38,221	8225800	353690	1,68E+07	5674300	0	182,92
P17918	P17918	Proliferating cell nuclear antigen	Pcna	3	0	4	3	3	0	4	3	26,1	28,785	2783900	0	5847900	1122200	0	42,393
Q9WU78	Q9WU78	Programmed cell death 6-interacting protein	Pdcd6ip	5	0	7	36	5	0	7	36	48,6	96,023	2590000	0	1,85E+07	3,84E+08	0	323,31
Q922R8	Q922R8	Protein disulfide-isomerase A6	Pdia6	2	1	2	3	2	1	2	3	10	48,1	1539100	152140	2042100	3317100	0	28,506
Q8K183	Q8K183	Pyridoxal kinase	Pdxk	1	0	1	1	1	0	1	1	8,7	35,015	1360200	0	1237000	994840	0	12,068
Q5SUR0	Q5SUR0	Phosphoribosylformylglycinamidine synthase	Pfas	1	0	3	3	1	0	3	3	5,2	144,63	687910	0	2116800	3625200	0	50,405
P12382;P47857	P12382	ATP-dependent 6-phosphofructokinase, liver type	Pfkl	2	0	5	9	1	0	4	7	18,7	85,359	2028600	0	8376800	1,43E+07	0	76,642
Q9WUA3	Q9WUA3	ATP-dependent 6-phosphofructokinase, platelet type	Pfkp	1	0	4	11	0	0	3	9	20,9	85,454	751790	0	3646100	3,35E+07	0	323,31
Q9DBJ1;O70250	Q9DBJ1	Phosphoglycerate mutase 1	Pgam1	0	0	3	3	0	0	3	3	27,6	28,832	0	0	3901400	5245400	0	29,909
Q9DCD0	Q9DCD0	6-phosphogluconate dehydrogenase, decarboxylating	Pgd	1	0	1	9	1	0	1	9	24,8	53,247	1203300	0	2349800	2,61E+07	0	162,41
P09411;P09041	P09411	Phosphoglycerate kinase 1	Pgk1	4	2	6	11	4	2	6	11	48,2	44,55	1,24E+07	787830	4,92E+07	5,38E+07	0	187,87
Q9CQ60	Q9CQ60	6-phosphogluconolactonase	Pgls	1	0	1	1	1	0	1	1	12,5	27,254	136390	0	286110	342320	0	12,238
Q61753	Q61753	D-3-phosphoglycerate dehydrogenase	Phgdh	7	2	10	22	7	2	10	22	41,8	56,585	4,57E+07	7697000	1,60E+08	3,77E+08	0	323,31
P52480;P53657	P52480	Pyruvate kinase PKM	Pkm	10	3	17	29	10	3	17	29	61,8	57,844	1,19E+08	5481300	6,48E+08	3,91E+08	0	323,31
P97350	P97350	Plakophilin-1	Pkp1	2	0	3	1	2	0	3	1	5,2	80,895	991960	0	1,25E+07	496130	0	59,601
P47713	P47713	Cytosolic phospholipase A2;Phospholipase A2;Lysophospholipase	Pla2g4a	1	0	1	1	1	0	1	1	2	85,221	228880	0	894290	331340	0,00096246	7,2355
Q8CIH5	Q8CIH5	1-phosphatidylinositol 4,5-bisphosphate phosphodiesterase gamma-2	Plcg2	4	0	6	7	4	0	6	7	7,6	147,59	3046200	0	8046300	8202400	0	95,155
Q9QXS1	Q9QXS1	Plectin	Plec	3	0	6	18	3	0	6	18	6	534,18	1427400	0	2758200	1,85E+07	0	173,93
Q9JHK5	Q9JHK5	Pleckstrin	Plek	0	0	1	5	0	0	1	5	24,6	39,9	0	0	974060	9995200	0	41,956
Q9CPS7	Q9CPS7	RNA-binding protein PNO1	Pno1	0	0	1	3	0	0	1	3	21	27,453	0	0	635340	1296200	0	23,694
P52432	P52432	DNA-directed RNA polymerases I and III subunit RPAC1	Polr1c	1	0	3	2	1	0	3	2	14,5	39,106	855270	0	4610100	480190	0	46,073
Q8CFI7	Q8CFI7	DNA-directed RNA polymerase II subunit RPB2	Polr2b	1	0	2	4	1	0	2	4	4,9	133,91	217260	0	992270	4269800	0	32,88
P17742	P17742	Peptidyl-prolyl cis-trans isomerase A;Peptidyl-prolyl cis-trans isomerase A, N-terminally processed	Ppia	0	0	2	1	0	0	2	1	25,6	17,971	0	0	1,25E+07	635750	0	47,848
P62137	P62137	Serine/threonine-protein phosphatase PP1-alpha catalytic subunit	Ppp1ca	1	0	3	4	1	0	2	1	18,8	37,54	76516	0	6404800	2217500	0	13,158
P62141	P62141	Serine/threonine-protein phosphatase PP1-beta catalytic subunit	Ppp1cb	2	0	3	4	2	0	2	2	14,4	37,186	824150	0	2555200	2222000	0	43,128
P63087	P63087	Serine/threonine-protein phosphatase PP1-gamma catalytic subunit	Ppp1cc	1	0	3	5	1	0	2	2	19,2	36,983	257590	0	4763300	8469200	0	48,262
P63330;P62715;P97470	P63330;P62715	Serine/threonine-protein phosphatase 2A catalytic subunit alpha isoform;Serine/threonine-protein phosphatase 2A catalytic subunit beta isoform	Ppp2ca;Ppp2cb	0	0	4	7	0	0	4	7	31,4	35,608	0	0	1,09E+07	4283400	0	231,5
Q76MZ3;Q7TNP2	Q76MZ3	Serine/threonine-protein phosphatase 2A 65 kDa regulatory subunit A alpha isoform	Ppp2r1a	3	1	4	1	3	1	4	1	9,7	65,322	2259900	85878	1,63E+07	700980	0	49,1
P1F6;Q6ZWR4;Q8BG02;Q92	Q6P1F6	Serine/threonine-protein phosphatase 2A 55 kDa regulatory subunit B alpha isoform	Ppp2r2a	2	1	4	3	2	1	4	3	17,2	51,691	2650700	56601	8008100	6000000	0	62,297
P35700	P35700	Peroxiredoxin-1	Prdx1	1	0	3	3	1	0	3	3	34,2	22,176	706460	0	1,89E+07	1380700	0	29,961
O08807	O08807	Peroxiredoxin-4	Prdx4	1	1	1	1	1	1	1	1	4	31,052	7483400	1386900	2,44E+07	2241500	0,0010373	8,2107
O08709	O08709	Peroxiredoxin-6	Prdx6	1	1	1	1	1	1	1	1	5,4	24,87	505050	202100	977930	292080	0,00089526	6,562
Q9QUR6	Q9QUR6	Prolyl endopeptidase	Prep	3	0	3	4	3	0	3	4	10,1	80,751	3942400	0	1,08E+07	5154300	0	65,473
P68181	P68181	cAMP-dependent protein kinase catalytic subunit beta	Prkacb	1	0	1	2	1	0	1	2	12,3	40,707	128440	0	1048100	1785400	0	18,725

Q9DBC7	Q9DBC7	cAMP-dependent protein kinase type I-alpha regulatory subunit;cAMP-dependent protein kinase type I-alpha regulatory subunit, N-terminally processed	Prkar1a	2	1	2	1	2	1	2	1	6,3	43,185	918170	166180	6757900	82481	0	20,943
Q9JIF0	Q9JIF0	Protein arginine N-methyltransferase 1	Prmt1	1	0	1	8	1	0	1	8	25,6	42,435	3338800	0	1,33E+07	1,29E+07	0	51,351
Q8CIG8	Q8CIG8	Protein arginine N-methyltransferase 5	Prmt5	3	0	4	3	3	0	4	3	11,1	72,679	5189800	0	1,15E+07	2833600	0	36,811
Q8CCF0	Q8CCF0	U4/U6 small nuclear ribonucleoprotein Prp31	Prpf31	2	0	2	1	2	0	2	1	5,6	55,429	426920	0	1,16E+07	1260300	0	16,844
Q9DAW6	Q9DAW6	U4/U6 small nuclear ribonucleoprotein Prp4	Prpf4	0	0	1	2	0	0	1	2	5,2	58,369	0	0	2547500	1717100	0	14,276
Q99PV0	Q99PV0	Pre-mRNA-processing-splicing factor 8	Prpf8	0	0	3	10	0	0	3	10	6	273,61	0	0	4648100	1,79E+07	0	73,828
Q9D7G0	Q9D7G0	Ribose-phosphate pyrophosphokinase 1	Prps1	0	0	3	5	0	0	1	2	34,6	34,834	0	0	5859600	6729900	0	55,227
Q61207	Q61207	Prosaposin	Psap	1	0	1	2	1	0	1	2	4,3	61,422	269710	0	262210	1599400	0	12,609
Q99K85	Q99K85	Phosphoserine aminotransferase	Psat1	5	1	2	11	5	1	2	11	36,2	40,472	5746700	160920	1,55E+07	2,55E+07	0	178,39
Q9R1P4	Q9R1P4	Proteasome subunit alpha type-1	Psma1	1	0	2	4	1	0	2	4	21,3	29,546	653250	0	823260	4327000	0	80,053
O70435	O70435	Proteasome subunit alpha type-3	Psma3	0	0	1	2	0	0	1	2	12,2	28,405	0	0	309820	1051200	0	29,802
Q9QUM9	Q9QUM9	Proteasome subunit alpha type-6	Psma6	0	0	1	1	0	0	1	1	5,3	27,372	0	0	261370	743050	0,0009542	7,1283
Q9Z2U0;Q9CWH6	Q9Z2U0;Q9CWH6	Proteasome subunit alpha type-7;Proteasome subunit alpha type-7-like	Psma7;Psma8	0	0	1	2	0	0	1	2	10,1	27,855	0	0	632380	1094400	0	11,625
P62192	P62192	26S protease regulatory subunit 4	Psmc1	2	0	4	6	2	0	4	6	23,2	49,184	403070	0	9926900	2,31E+07	0	55,592
P46471	P46471	26S protease regulatory subunit 7	Psmc2	7	2	8	12	7	2	8	12	42	48,647	4427700	97900	2,59E+07	4,77E+07	0	126,57
O88685	O88685	26S protease regulatory subunit 6A	Psmc3	1	0	2	6	1	0	2	6	18,6	49,548	654100	0	1008400	9112000	0	44,658
P54775	P54775	26S protease regulatory subunit 6B	Psmc4	3	1	2	6	3	1	2	6	16,5	47,408	2202800	168140	3586500	1,06E+07	0	36,247
P62196	P62196	26S protease regulatory subunit 8	Psmc5	1	0	1	6	1	0	1	6	21,2	45,626	312840	0	1892000	6878400	0	38,4
P62334	P62334	26S protease regulatory subunit 10B	Psmc6	3	1	7	8	3	1	7	8	32,4	44,172	3333500	144540	1,20E+07	1,51E+07	0	73,25
Q3TXS7	Q3TXS7	26S proteasome non-ATPase regulatory subunit 1	Psmd1	3	0	5	8	3	0	5	8	16,1	105,73	4823500	0	2,17E+07	7973800	0	67,666
Q8BG32	Q8BG32	26S proteasome non-ATPase regulatory subunit 11	Psmd11	1	1	2	7	1	1	2	7	26,5	47,436	3719000	1219800	1,12E+07	2,90E+07	0	303,34
O35593	O35593	26S proteasome non-ATPase regulatory subunit 14	Psmd14	1	1	1	2	1	1	1	2	8,7	34,577	672150	87971	1919500	6229600	0	11,624
Q8VDM4	Q8VDM4	26S proteasome non-ATPase regulatory subunit 2	Psmd2	2	0	4	12	2	0	4	12	21,8	100,2	903990	0	1,40E+07	5,49E+07	0	99,918
P14685	P14685	26S proteasome non-ATPase regulatory subunit 3	Psmd3	1	0	3	12	1	0	3	12	31,5	60,718	861210	0	6089800	4,47E+07	0	161,46
Q8BJY1	Q8BJY1	26S proteasome non-ATPase regulatory subunit 5	Psmd5	1	0	1	4	1	0	1	4	9,9	55,971	2228300	0	140740	3699000	0	32,641
Q99JI4	Q99JI4	26S proteasome non-ATPase regulatory subunit 6	Psmd6	2	0	3	12	2	0	3	12	40,6	45,536	1410500	0	5070800	1,67E+07	0	86,782
P61290	P61290	Proteasome activator complex subunit 3	Psme3	0	0	1	2	0	0	1	2	15,4	29,506	0	0	686880	1559200	0	32,268
Q5SSW2	Q5SSW2	Proteasome activator complex subunit 4	Psme4	0	0	1	1	0	0	1	1	0,8	211,19	0	0	573200	805700	0,00099502	7,492
Q9JK23	Q9JK23	Proteasome assembly chaperone 1	Psmg1	0	0	1	1	0	0	1	1	9,3	33,104	0	0	480510	1301100	0	11,869
P17225	P17225	Polypyrimidine tract-binding protein 1	Ptbp1	1	0	1	3	1	0	1	3	11,2	56,477	39398	0	4033100	3351800	0	24,327
P29351	P29351	Tyrosine-protein phosphatase non-receptor type 6	Ptpn6	6	1	8	12	6	1	8	12	30,9	67,558	9844700	20996	4,12E+07	4,06E+07	0	130,91
P18052	P18052	Receptor-type tyrosine-protein phosphatase alpha	Ptpra	0	0	1	2	0	0	1	2	2,9	93,696	0	0	605300	1517000	0	11,567
P06800	P06800	Receptor-type tyrosine-protein phosphatase C	Ptprc	0	0	2	7	0	0	2	7	5,2	144,6	0	0	2880600	1,58E+07	0	96,6
O35295	O35295	Transcriptional activator protein Pur-beta	Purb	1	0	2	2	1	0	2	2	12,3	33,901	76220	0	1747700	417710	0	19,44
Q8CI94;Q9WUB3;Q9ET01	Q8CI94	Glycogen phosphorylase, brain form	Pygb	0	0	1	3	0	0	1	3	6,8	96,729	0	0	1417200	3718100	0	26,311
Q8BML9	Q8BML9		Qars	5	1	6	10	5	1	6	10	19,7	87,676	5099900	179070	3,59E+07	1,49E+07	0	95,504
P62492;P46638	P62492;P46638	Ras-related protein Rab-11A;Ras-related protein Rab-11B	Rab11a;Rab11b	0	0	1	4	0	0	1	4	20,8	24,393	0	0	422270	2989500	0	24,258
Q9CQD1	Q9CQD1	Ras-related protein Rab-5A	Rab5a	0	0	1	3	0	0	1	2	17,2	23,598	0	0	424770	1014800	0	11,64
P35278	P35278	Ras-related protein Rab-5C	Rab5c	0	0	1	2	0	0	1	1	10,6	23,412	0	0	1999800	1541700	0,00089686	6,5663
P61294;P35279;Q8BHD0	P61294;P35279;Q8BHD0	Ras-related protein Rab-6B;Ras-related protein Rab-6A;Ras-related protein Rab-39A	Rab6b;Rab6a;Rab39a	0	0	1	2	0	0	1	2	11,1	23,461	0	0	1625100	8057500	0	39,41
P51150	P51150	Ras-related protein Rab-7a	Rab7a	1	0	2	7	1	0	2	7	40,1	23,489	394590	0	4260500	1,16E+07	0	323,31
P63001;P60764	P63001;P60764	Ras-related C3 botulinum toxin substrate 1;Ras-related C3 botulinum toxin substrate 3	Rac1;Rac3	2	0	4	7	0	0	1	3	29,7	21,45	485190	0	2433100	2,52E+07	0	44,138
Q8C570	Q8C570	mRNA export factor	Rae1	1	0	2	1	1	0	2	1	11,1	40,965	229290	0	1418800	819520	0	88,311
Q64012	Q64012	RNA-binding protein Raly	Raly	1	0	3	4	1	0	3	4	17,3	33,188	279290	0	3603000	3946400	0	27,045
P62827;Q61820	P62827	GTP-binding nuclear protein Ran	Ran	4	1	4	9	4	1	4	9	35,2	24,423	3949200	611760	3,20E+07	1,19E+08	0	69,716
P46061	P46061	Ran GTPase-activating protein 1	Rangap1	1	0	2	2	1	0	2	2	7,6	63,53	274440	0	4102300	1245700	0	65,367
Q9D0I9	Q9D0I9	Arginine--tRNA ligase, cytoplasmic	Rars	5	2	9	14	5	2	9	14	34,4	75,673	1,34E+07	736170	5,81E+07	5,27E+07	0	226,46
Q8VH51	Q8VH51	RNA-binding protein 39	Rbm39	2	0	4	2	2	0	4	2	12,6	59,406	370500	0	1,57E+07	2639600	0	74,425
Q8VE37	Q8VE37	Regulator of chromosome condensation	Rcc1	1	0	2	4	1	0	2	4	21,4	44,93	3437700	0	8567500	1,04E+07	0	38,799
Q8BK67	Q8BK67	Protein RCC2	Rcc2	3	0	3	7	3	0	3	7	20,2	55,983	3190900	0	9517100	2,49E+07	0	68,195
Q9JJT0	Q9JJT0	RNA 3-terminal phosphate cyclase-like protein	Rcl1	0	0	1	2	0	0	1	2	8,3	40,84	0	0	870820	1022100	0	35,696
Q9WUK4	Q9WUK4	Replication factor C subunit 2	Rfc2	0	0	1	2	0	0	1	2	7,4	38,724	0	0	1785700	1082500	0	13,685
Q8R323	Q8R323	Replication factor C subunit 3	Rfc3	1	1	1	1	1	1	1	1	3,9	40,526	119640	24071	769210	314660	0,001065	10,055
Q9D0F6	Q9D0F6	Replication factor C subunit 5	Rfc5	1	0	1	3	1	0	1	3	12,1	38,096	340770	0	1900200	890570	0	24,017
Q9QUI0	Q9QUI0	Transforming protein RhoA	Rhoa	2	0	2	5	1	0	1	1	23,8	21,782	733510	0	2878100	7830300	0	33,051
Q62159	Q62159	Rho-related GTP-binding protein RhoC	Rhoc	1	0	2	5	0	0	1	1	23,8	22,006	0	0	315850	1438400	0,00090744	6,6388
E9Q555	E9Q555	E3 ubiquitin-protein ligase RNF213	Rnf213	3	1	11	35	3	1	11	35	8,7	584,78	1593300	382190	9779400	5,34E+07	0	323,31
Q91VI7	Q91VI7	Ribonuclease inhibitor	Rnh1	1	1	1	3	1	1	1	3	12,1	49,816	758740	125670	2845700	2480900	0	30,668
Q9D0L8	Q9D0L8	mRNA cap guanine-N7 methyltransferase	Rnmt	1	0	2	1	1	0	2	1	9,9	53,291	204620	0	2008400	89317	0	18,151
Q8VCT3	Q8VCT3	Aminopeptidase B	Rnpep	1	1	2	1	1	1	2	1	6,8	72,415	868760	40711	3724000	539090	0	21,829
Q9JJ80	Q9JJ80	Ribosome production factor 2 homolog	Rpf2	0	0	1	2	0	0	1	2	11,8	35,363	0	0	488150	2130500	0	47,69
Q6ZWV3;P86048	Q6ZWV3;P86048	60S ribosomal protein L10;60S ribosomal protein L10-like	Rpl10;Rpl10l	2	0	7	9	2	0	7	9	37,9	24,604	1577700	0	2,48E+08	5,12E+07	0	85,759
P53026	P53026	60S ribosomal protein L10a	Rpl10a	1	1	3	7	1	1	3	7	30,4	24,916	2638000	991160	9,04E+07	4,12E+07	0	57,802
Q9CXW4	Q9CXW4	60S ribosomal protein L11	Rpl11	1	0	2	3	1	0	2	3	18,5	20,252	320430	0	1302000	2,40E+07	0	59,413
P35979	P35979	60S ribosomal protein L12	Rpl12	5	0	6	5	5	0	6	5	54,5	17,804	3054800	0	4,56E+07	5,17E+07	0	46,819
P47963	P47963	60S ribosomal protein L13	Rpl13	6	4	7	8	6	4	7	8	30,8	24,305	1,85E+07	513370	4,02E+08	9,06E+07	0	139,23
P19253	P19253	60S ribosomal protein L13a	Rpl13a	2	2	7	13	2	2	7	13	47,3	23,464	4594600	382110	8,40E+07	1,16E+08	0	121,52
Q9CR57	Q9CR57	60S ribosomal protein L14	Rpl14	3	2	5	7	3	2	5	7	31,8	23,564	2789600	680740	1,12E+08	1,27E+08	0	244,38
Q9CZM2	Q9CZM2	60S ribosomal protein L15	Rpl15	1	1	2	4	1	1	2	4	14,7	24,146	7042900	740240	1,67E+08	1,05E+07	0	23,762
Q9CPR4	Q9CPR4	60S ribosomal protein L17	Rpl17	0	0	2	7	0	0	2	7	34,2	21,423	0	0	3739000	8781100	0	48,408
P35980	P35980	60S ribosomal protein L18	Rpl18	4	3	8	8	4	3	8	8	35,1	21,644	5,65E+07	3867000	4,60E+08	1,40E+08	0	323,31
P62717	P62717	60S ribosomal protein L18a	Rpl18a	4	2	6	7	4	2	6	7	41,5	20,732	1,44E+07	566690	1,37E+08	3,53E+07	0	144,26
P84099	P84099	60S ribosomal protein L19	Rpl19	0	0	3	6	0	0	3	6	23,5	23,466	0	0	4782200	2,36E+07	0	84,968
O09167	O09167	60S ribosomal protein L21	Rpl21	2	0	2	4	2	0	2	4	36,9	18,562	732820	0	3,67E+07	1,38E+07	0	29,469
P67984	P67984	60S ribosomal protein L22	Rpl22	2	0	2	4	2	0	2	4	31,2	14,759	2501400	0	1,37E+07	2,02E+07	0	98,479
Q9D7S7	Q9D7S7	60S ribosomal protein L22-like 1	Rpl22l1	0	0	1	2	0	0	1	2	22,1	14,467	0	0	623920	1129200	0	12,908

P62830	P62830	60S ribosomal protein L23	Rpl23	3	0	5	5	3	0	5	5	40	14,865	1001300	0	3,36E+07	1,04E+07	0	74,665
P62751	P62751	60S ribosomal protein L23a	Rpl23a	1	0	1	1	1	0	1	1	8,3	17,695	1140100	0	6749000	1778300	0,0033727	6,3445
Q8BP67	Q8BP67	60S ribosomal protein L24	Rpl24	2	0	5	6	2	0	5	6	26,8	17,779	4024600	0	3,31E+07	3,49E+07	0	76,182
P61255	P61255	60S ribosomal protein L26	Rpl26	2	0	2	2	2	0	2	2	14,5	17,258	828440	0	4383400	4976700	0	11,591
P61358	P61358	60S ribosomal protein L27	Rpl27	1	0	2	4	1	0	2	4	25	15,798	232610	0	2,15E+07	1,51E+07	0	25,413
P14115	P14115	60S ribosomal protein L27a	Rpl27a	1	0	4	2	1	0	4	2	23	16,605	119570	0	7414600	1439800	0	24,311
P41105	P41105	60S ribosomal protein L28	Rpl28	1	0	1	4	1	0	1	4	35	15,733	264400	0	2,94E+07	1,18E+07	0	24,608
P27659	P27659	60S ribosomal protein L3	Rpl3	3	1	3	14	3	1	3	14	31,8	46,109	4,33E+07	146380	2,61E+08	3,55E+08	0	231,41
P62889	P62889	60S ribosomal protein L30	Rpl30	2	1	2	5	2	1	2	5	51,3	12,784	4568800	260030	3,97E+07	1,27E+07	0	75,752
P62900	P62900	60S ribosomal protein L31	Rpl31	1	2	2	2	1	2	2	2	15,2	14,463	1767400	374240	5,23E+07	8110800	0	33,023
P62911	P62911	60S ribosomal protein L32	Rpl32	0	0	2	3	0	0	2	3	27,4	15,86	0	0	1421400	5103800	0	72,388
Q6ZWV7	Q6ZWV7	60S ribosomal protein L35	Rpl35	2	0	2	3	2	0	2	3	22	14,552	1235400	0	1,18E+07	1,10E+07	0	73,36
P47964	P47964	60S ribosomal protein L36	Rpl36	2	1	2	1	2	1	2	1	13,3	12,215	1548100	163330	7,88E+07	3186900	0	11,366
Q9D8E6	Q9D8E6	60S ribosomal protein L4	Rpl4	9	1	16	23	9	1	16	23	49,9	47,153	1,18E+07	30540	1,61E+08	5,77E+08	0	323,31
P47962	P47962	60S ribosomal protein L5	Rpl5	3	0	2	10	3	0	2	10	34,3	34,4	1,04E+07	0	1,57E+07	1,36E+07	0	61,404
P47911	P47911	60S ribosomal protein L6	Rpl6	4	2	8	9	4	2	8	9	34,5	33,509	2,81E+07	774000	2,13E+08	2,89E+08	0	246,63
P14148	P14148	60S ribosomal protein L7	Rpl7	3	2	10	15	3	2	10	15	44,8	31,419	1,75E+07	1407200	4,87E+08	1,86E+08	0	130,81
P12970	P12970	60S ribosomal protein L7a	Rpl7a	4	1	10	14	4	1	10	14	39,8	29,976	2664700	41590	1,54E+08	2,74E+08	0	205,17
P62918	P62918	60S ribosomal protein L8	Rpl8	2	0	4	6	2	0	4	6	21,8	28,024	4458400	0	1,82E+08	6,92E+07	0	97,363
P51410	P51410	60S ribosomal protein L9	Rpl9	1	0	1	7	1	0	1	7	33,9	21,881	193200	0	1708500	1,31E+07	0	43,761
P14869	P14869	60S acidic ribosomal protein P0	Rplp0	6	2	9	12	6	2	9	12	53	34,216	4530600	162640	8,51E+07	1,59E+08	0	195,93
P63325	P63325	40S ribosomal protein S10	Rps10	1	0	1	3	1	0	1	3	14,5	18,916	319420	0	1542500	3384900	0	18,907
P62281	P62281	40S ribosomal protein S11	Rps11	8	0	9	15	8	0	9	15	70,3	18,431	7040300	0	6,99E+07	3,64E+07	0	100,01
P62301	P62301	40S ribosomal protein S13	Rps13	2	2	3	5	2	2	3	5	34,4	17,222	3,25E+07	1833600	1,40E+08	6,12E+07	0	53,142
P62264	P62264	40S ribosomal protein S14	Rps14	5	1	7	8	5	1	7	8	40,4	16,273	1,22E+07	401450	8,57E+07	3,72E+07	0	102,56
P62245	P62245	40S ribosomal protein S15a	Rps15a	1	0	3	5	1	0	3	5	38,5	14,839	3403800	0	1,18E+07	2,68E+07	0	33,138
P14131	P14131	40S ribosomal protein S16	Rps16	6	2	8	9	6	2	8	9	54,1	16,445	6,71E+07	2012200	1,37E+08	4,75E+07	0	107,58
P63276	P63276	40S ribosomal protein S17	Rps17	1	0	3	3	1	0	3	3	16,3	15,524	624490	0	5902600	8173300	0	22,373
P62270	P62270	40S ribosomal protein S18	Rps18	7	3	10	9	7	3	10	9	48,7	17,718	8,30E+07	1356800	3,58E+08	4,02E+07	0	242,12
Q9CZX8	Q9CZX8	40S ribosomal protein S19	Rps19	2	0	4	5	2	0	4	5	29	16,085	753870	0	7931300	1,40E+07	0	121,96
P25444	P25444	40S ribosomal protein S2	Rps2	3	0	7	11	3	0	7	11	40,3	31,231	1,10E+07	0	6,16E+07	2,84E+08	0	169,92
P60867	P60867	40S ribosomal protein S20	Rps20	1	0	2	3	1	0	2	3	25,2	13,373	396970	0	2,01E+07	1,40E+07	0	111,79
P62267	P62267	40S ribosomal protein S23	Rps23	1	0	2	2	1	0	2	2	16,1	15,807	156000	0	2655300	5004000	0	39,056
P62849	P62849	40S ribosomal protein S24	Rps24	1	1	3	4	1	1	3	4	34,6	15,423	1,50E+07	382900	6,12E+07	1,96E+07	0	53,42
P62852	P62852	40S ribosomal protein S25	Rps25	2	1	2	4	2	1	2	4	24	13,742	5753400	131020	4,40E+07	3,61E+07	0	43,216
P62855	P62855	40S ribosomal protein S26	Rps26	3	1	3	3	3	1	3	3	31,3	13,015	7,66E+07	2572300	2,70E+08	2826700	0	33,596
Q6ZWU9;Q6ZWY3	Q6ZWU9;Q6ZWY3	40S ribosomal protein S27;40S ribosomal protein S27-like	Rps27;Rps27l	1	0	2	2	1	0	2	2	39,3	9,461	2430100	0	2614700	392070	0	18,645
2983;P62984;P0CG49;P0CG	2983;P62984;P0CG49;P0CG	Ubiquitin-40S ribosomal protein S27a;Ubiquitin;40S ribosomal protein S27a;Ubiquitin-60S ribosomal protein L40;Ubiquitin;60S ribosomal protein L40;Polyubiquitin-B;Ubiquitin;Polyubiquitin-C;Ubiquitin;Ubiquitin-related 1;Ubiquitin-related 2	ps27a;Uba52;Ubb;Ub	1	0	6	6	1	0	6	6	35,3	17,951	1784500	0	3,11E+07	2,18E+08	0	211,26
P62908	P62908	40S ribosomal protein S3	Rps3	8	4	10	16	8	4	10	16	71,6	26,674	2,64E+07	1111200	3,32E+08	2,60E+08	0	290,71
P97351	P97351	40S ribosomal protein S3a	Rps3a	4	0	8	15	4	0	8	15	56,4	29,885	3383100	0	7,48E+07	1,63E+08	0	323,31
P62702	P62702	40S ribosomal protein S4, X isoform	Rps4x	6	0	14	15	6	0	14	15	57,8	29,597	4507200	0	1,76E+08	1,58E+08	0	236,55
P97461	P97461	40S ribosomal protein S5;40S ribosomal protein S5, N-terminally processed	Rps5	2	0	4	7	2	0	4	7	22,1	22,889	1088600	0	2,97E+07	2,36E+07	0	123,47
P62754	P62754	40S ribosomal protein S6	Rps6	4	1	5	9	4	1	5	9	28,1	28,68	3185700	64654	1,86E+07	2,73E+08	0	210,63
P18654	P18654	Ribosomal protein S6 kinase alpha-3	Rps6ka3	1	0	1	1	1	0	1	1	3,1	83,693	197160	0	1205100	693230	0	11,611
P62082	P62082	40S ribosomal protein S7	Rps7	0	0	2	4	0	0	2	4	23,7	22,127	0	0	870800	4442600	0	23,293
P62242	P62242	40S ribosomal protein S8	Rps8	5	2	8	10	5	2	8	10	47,1	24,205	2,25E+07	685880	3,09E+08	2,13E+08	0	288,92
Q6ZWN5	Q6ZWN5	40S ribosomal protein S9	Rps9	6	4	12	11	6	4	12	11	34,5	22,591	2,34E+07	2315300	3,88E+08	9,08E+07	0	119,1
P14206	P14206	40S ribosomal protein SA	Rpsa	7	4	8	11	7	4	8	11	42	32,838	1,20E+08	4105900	2,72E+08	8,17E+07	0	323,31
Q99PL5	Q99PL5	Ribosome-binding protein 1	Rrbp1	1	0	2	1	1	0	2	1	1,4	172,88	64869	0	846590	1906300	0	13,49
Q6P5B0	Q6P5B0	RRP12-like protein	Rrp12	1	0	2	2	1	0	2	2	2	143,13	170330	0	4070600	2541700	0	16,179
Q8BVY0	Q8BVY0	Ribosomal L1 domain-containing protein 1	Rsl1d1	0	0	1	9	0	0	1	9	24,1	50,421	0	0	3321800	2,93E+07	0	266,06
Q99LF4	Q99LF4	tRNA-splicing ligase RtcB homolog	Rtcb	3	0	3	4	3	0	3	4	14,9	55,249	1760400	0	6299500	9675700	0	37,024
Q99P72	Q99P72	Reticulon-4	Rtn4	0	0	1	4	0	0	1	4	5,2	126,61	0	0	482420	8027600	0	25,688
P60122	P60122	RuvB-like 1	Ruvbl1	2	2	4	10	2	2	4	10	32,7	50,213	913810	161350	6287000	2,00E+07	0	95,615
Q9WTM5	Q9WTM5	RuvB-like 2	Ruvbl2	6	0	7	7	6	0	7	7	27,9	51,112	7792300	0	1,58E+07	1,64E+07	0	210,82
P04918	P04918	Serum amyloid A-3 protein	Saa3	0	0	1	1	0	0	1	1	8,2	13,773	0	0	256490	3622300	0,002584	6,4121
Q60710	Q60710	Deoxynucleoside triphosphate triphosphohydrolase SAMHD1	Samhd1	1	0	1	5	1	0	1	5	8,3	72,65	1012000	0	9752100	7768900	0	63,191
P26638	P26638	Serine--tRNA ligase, cytoplasmic	Sars	5	2	7	13	5	2	7	13	32,6	58,388	7222400	573750	4,21E+07	8,42E+07	0	236,01
Q689Z5	Q689Z5	Protein strawberry notch homolog 1	Sbno1	0	0	2	2	0	0	1	1	1,9	153,74	0	0	1391100	645720	0	13,507
O35609	O35609	Secretory carrier-associated membrane protein 3	Scamp3	3	2	2	6	3	2	2	6	19,8	38,458	1349400	218540	597460	1,62E+07	0	96,441
O08992	O08992	Syntenin-1	Sdcbp	0	0	2	5	0	0	2	5	16,7	32,379	0	0	3111500	1,04E+07	0	49,488
O08547	O08547	Vesicle-trafficking protein SEC22b	Sec22b	1	0	1	1	1	0	1	1	6,5	24,74	269750	0	742530	237940	0,00098135	7,3146
Q9D662	Q9D662	Protein transport protein Sec23B	Sec23b	1	0	1	2	1	0	1	2	3,3	86,436	459100	0	687950	2284300	0	14,661
Q3UPL0	Q3UPL0	Protein transport protein Sec31A	Sec31a	2	0	4	4	2	0	4	4	7,8	133,57	2224900	0	7239800	4319000	0	54,24
P97364	P97364	Selenide, water dikinase 2	Sephs2	1	1	1	1	1	1	1	1	4,6	47,833	59841	7042,1	63507	71535	0,001007	7,6146
P42208	P42208	Septin-2	Sept 2	2	0	2	3	2	0	2	3	15,5	41,525	462860	0	4138100	3172900	0	52,062
Q921M3	Q921M3	Splicing factor 3B subunit 3	Sf3b3	2	0	3	5	2	0	3	5	5,7	135,55	725540	0	6587000	1,26E+07	0	44,492
Q8VIJ6	Q8VIJ6	Splicing factor, proline- and glutamine-rich	Sfpq	4	1	4	8	3	1	3	7	15,9	75,441	1,32E+07	80504	3,39E+07	3,40E+07	0	65,828
P50431	P50431	Serine hydroxymethyltransferase, cytosolic	Shmt1	1	0	2	4	1	0	2	4	12,8	52,6	483060	0	5656000	9075600	0	40,397
P53986	P53986	Monocarboxylate transporter 1	Slc16a1	0	1	2	6	0	1	2	6	12,6	53,267	0	121890	725600	4,78E+07	0	139,5
P51912	P51912	Neutral amino acid transporter B(0)	Slc1a5	0	0	1	8	0	0	1	8	17,4	58,482	0	0	1014800	6,79E+07	0	106,91
P10852	P10852	4F2 cell-surface antigen heavy chain	Slc3a2	4	0	5	7	4	0	5	7	15,4	58,336	6269600	0	2,40E+07	4,76E+07	0	74,122
Q9Z127	Q9Z127	Large neutral amino acids transporter small subunit 1	Slc7a5	2	1	3	5	2	1	3	4	8	55,872	643680	76088	3220500	2,31E+07	0	33,859
Q3TKT4	Q3TKT4	Transcription activator BRG1	Smarca4	0	0	1	1	0	0	1	1	1,7	181,43	0	0	1698500	510910	0	12,137

Q91ZW3;Q6PGB8	Q91ZW3;Q6PGB8	SWI/SNF-related matrix-associated actin-dependent regulator of chromatin subfamily A member 5;Probable global transcription activator SNF2L1	Smarca5;Smarca1	0	0	3	7	0	0	3	7	8,4	121,63	0	0	7969500	8907900	0	55,394
Q9CU62	Q9CU62	Structural maintenance of chromosomes protein 1A	Smc1a	0	0	1	9	0	0	1	9	7,7	143,23	0	0	67342	9521000	0	64,393
Q8CG48	Q8CG48	Structural maintenance of chromosomes protein 2	Smc2	1	0	1	13	1	0	1	13	13,9	134,24	143260	0	769100	1,94E+07	0	117,67
P58242	P58242	Acid sphingomyelinase-like phosphodiesterase 3b	Smpdl3b	1	1	2	2	1	1	2	2	10,1	51,599	321880	112740	4771100	6534500	0	19,38
O09044	O09044	Synaptosomal-associated protein 23	Snap23	0	0	1	2	0	0	1	2	12,4	23,261	0	0	161240	633920	0	12,694
Q78PY7	Q78PY7	Staphylococcal nuclease domain-containing protein 1	Snd1	9	1	8	10	9	1	8	10	20,2	102,09	1,25E+07	514070	2,54E+07	3,67E+07	0	323,31
Q6P4T2	Q6P4T2	U5 small nuclear ribonucleoprotein 200 kDa helicase	Snrnp200	1	0	8	17	1	0	8	17	11,4	244,54	497730	0	2,54E+07	3,38E+07	0	183,49
Q62189	Q62189	U1 small nuclear ribonucleoprotein A	Snrpa	1	1	1	1	1	1	1	1	4,2	31,835	1485600	306700	2061600	2791600	0,0058285	6,3096
P27048;P63163	P27048;P63163	Small nuclear ribonucleoprotein-associated protein B;Small nuclear ribonucleoprotein-associated protein N	Snrpb;Snrpn	1	0	2	3	1	0	2	3	16	23,656	1010800	0	1,08E+07	1404500	0	44,309
P62317	P62317	Small nuclear ribonucleoprotein Sm D2	Snrpd2	1	0	1	1	1	0	1	1	8,5	13,527	300240	0	1127400	487800	0	29,937
P62320	P62320	Small nuclear ribonucleoprotein Sm D3	Snrpd3	2	1	2	2	2	1	2	2	15,1	13,916	1565400	147830	1,14E+07	2986600	0	81,895
Q9CWK8;Q9WV80	Q9CWK8	Sorting nexin-2	Snx2	2	1	2	3	2	1	2	3	7,7	58,47	4734600	319720	3878300	5382400	0	27,056
Q9D8U8	Q9D8U8	Sorting nexin-5	Snx5	1	0	1	3	1	0	1	3	11,4	46,797	217160	0	367200	9333400	0	32,629
Q64105	Q64105	Sepiapterin reductase	Spr	3	2	6	6	3	2	6	6	40,2	27,883	979410	290400	1,10E+07	3976100	0	247,99
Q64674	Q64674	Spermidine synthase	Srm	3	2	3	4	3	2	3	4	17,5	33,995	4641800	358840	1,50E+07	3265700	0	39,217
Q8BMA6	Q8BMA6	Signal recognition particle subunit SRP68	Srp68	0	0	1	4	0	0	1	4	8,2	70,573	0	0	5449200	3885500	0	130,93
O70551	O70551	SRSF protein kinase 1	Srpk1	0	0	1	2	0	0	1	2	6	73,088	0	0	1822400	1101500	0	20,036
Q99MR6	Q99MR6	Serrate RNA effector molecule homolog	Srrt	4	0	4	9	4	0	4	9	10,9	100,45	3791000	0	1,83E+07	1,66E+07	0	86,212
Q6PDM2	Q6PDM2	Serine/arginine-rich splicing factor 1	Srsf1	3	1	7	7	2	1	6	7	43,5	27,744	577120	35034	9,31E+07	1,41E+07	0	64,709
Q62093	Q62093	Serine/arginine-rich splicing factor 2	Srsf2	1	0	4	1	1	0	4	1	19,5	25,476	1189000	0	9,49E+07	1313400	0	152,17
P84104	P84104	Serine/arginine-rich splicing factor 3	Srsf3	1	0	3	2	1	0	2	1	18,3	19,329	2190700	0	7,68E+07	3091700	0	16,135
Q3TWW8;Q8VE97	Q3TWW8;Q8VE97	Serine/arginine-rich splicing factor 6;Serine/arginine-rich splicing factor 4	Srsf6;Srsf4	2	0	2	1	1	0	1	1	5,3	39,025	3498800	0	9,27E+07	218810	0	26,41
Q8BL97	Q8BL97	Serine/arginine-rich splicing factor 7	Srsf7	0	0	6	4	0	0	5	3	27,3	30,817	0	0	3,84E+07	3247100	0	41,837
Q9D0B0	Q9D0B0	Serine/arginine-rich splicing factor 9	Srsf9	1	0	3	1	0	0	2	1	12,6	25,661	0	0	2532700	244530	0	12,782
P32067	P32067	Lupus La protein homolog	Ssb	0	0	3	4	0	0	3	4	16,1	47,756	0	0	8139400	1,12E+07	0	46,364
Q08943	Q08943	FACT complex subunit SSRP1	Ssrp1	0	0	2	1	0	0	2	1	3,2	80,859	0	0	2019300	450550	0	20,27
Q9JI11;Q9JI10	Q9JI11;Q9JI10	Serine/threonine-protein kinase 4;Serine/threonine-protein kinase 4 37kDa subunit;Serine/threonine-protein kinase 4 18kDa subunit;Serine/threonine-protein kinase 3;Serine/threonine-protein kinase 3 36kDa subunit;Serine/threonine-protein kinase 3 20kDa subunit	Stk4;Stk3	0	0	1	1	0	0	1	1	5,5	55,541	0	0	1093100	280350	0	11,438
Q9Z1Z2	Q9Z1Z2	Serine-threonine kinase receptor-associated protein	Strap	0	0	1	2	0	0	1	2	12,9	38,442	0	0	3105100	2610200	0	19,318
Q64324	Q64324	Syntaxin-binding protein 2	Stxbp2	1	0	1	3	1	0	1	3	7,8	66,357	180750	0	1289000	3559200	0	35,143
Q8BR65	Q8BR65	Sin3 histone deacetylase corepressor complex component SDS3	Suds3	0	1	1	1	0	1	1	1	5,8	38,107	0	513580	1338300	859740	0,0033473	6,3213
Q920B9	Q920B9	FACT complex subunit SPT16	Supt16h	0	0	3	6	0	0	3	6	7,5	119,82	0	0	4020500	7324700	0	51,757
Q7TMK9	Q7TMK9	Heterogeneous nuclear ribonucleoprotein Q	Syncrip	2	1	2	1	2	1	2	1	5,8	69,632	3836000	410960	1,92E+07	3364900	0	25,24
O55100	O55100	Synaptogyrin-1	Syngr1	0	0	1	2	0	0	1	2	10,3	25,652	0	0	552560	1415300	0	12,203
O55101	O55101	Synaptogyrin-2	Syngr2	0	0	1	2	0	0	1	2	12,9	24,778	0	0	710240	2419000	0	11,713
Q9WVA4;Q9R1Q8	Q9WVA4	Transgelin-2	Tagln2	4	3	4	5	4	3	4	5	31,7	22,395	9060100	2065000	1,06E+07	1727200	0	60,684
Q921F2	Q921F2	TAR DNA-binding protein 43	Tardbp	0	0	1	2	0	0	1	2	7,2	44,547	0	0	1753700	1492000	0	14,964
Q9D0R2	Q9D0R2	Threonine--tRNA ligase, cytoplasmic	Tars	4	1	3	9	4	1	3	9	18,8	83,355	4295400	43060	1,34E+07	1,76E+07	0	97,792
Q8BYA0	Q8BYA0	Tubulin-specific chaperone D	Tbcd	4	2	4	2	4	2	4	2	6,5	133,32	1363500	31652	6284300	1192300	0	41,998
Q8C4J7	Q8C4J7	Transducin beta-like protein 3	Tbl3	1	0	1	5	1	0	1	5	7,9	88,265	58082	0	135990	3763300	0	39,954
P11983	P11983	T-complex protein 1 subunit alpha	Tcp1	10	4	10	18	10	4	10	18	41	60,448	7,66E+07	3933500	1,57E+08	8,47E+07	0	310,13
Q3URQ0	Q3URQ0	Testis-expressed sequence 10 protein	Tex10	0	0	1	1	0	0	1	1	1,5	105,21	0	0	478360	176500	0,00093023	6,8378
Q62351	Q62351	Transferrin receptor protein 1	Tfrc	0	0	1	11	0	0	1	11	21,2	85,73	0	0	566650	5,52E+07	0	183,75
Q5U4D9	Q5U4D9	THO complex subunit 6 homolog	Thoc6	1	0	2	1	1	0	2	1	10,6	37,314	186120	0	871470	276670	0	14,702
P70318;P52912	P70318;P52912	Nucleolysin TIAR;Nucleolysin TIA-1	Tial1;Tia1	0	0	1	1	0	0	1	1	6,1	43,388	0	0	1129700	327680	0	14,043
Q8BZA9	Q8BZA9	Fructose-2,6-bisphosphatase TIGAR	Tigar	0	0	1	1	0	0	1	1	6,3	29,19	0	0	467790	504960	0,0010753	10,704
P40142	P40142	Transketolase	Tkt	1	0	2	10	1	0	2	10	24,2	67,63	1,48E+07	0	1,01E+07	2,03E+07	0	67,474
P26039;Q71LX4	P26039	Talin-1	Tln1	1	0	5	25	1	0	5	25	13,6	269,82	562560	0	1990300	4,17E+07	0	323,31
Q9CYG7	Q9CYG7	Mitochondrial import receptor subunit TOM34	Tomm34	1	1	1	4	1	1	1	4	19,1	34,278	471280	182190	5004800	3981400	0	47,632
Q01320	Q01320	DNA topoisomerase 2-alpha	Top2a	0	0	1	13	0	0	1	10	10,5	172,79	0	0	1084100	2,28E+07	0	323,31
Q99PW4	Q99PW4	TP53-regulating kinase	Tp53rk	1	0	2	1	1	0	2	1	13,1	27,393	258720	0	3010000	276220	0	14,221
P17751	P17751	Triosephosphate isomerase	Tpi1	1	2	2	8	1	2	2	8	27,8	32,191	405830	276280	3442600	1,08E+07	0	69,244
Q64514	Q64514	Tripeptidyl-peptidase 2	Tpp2	1	0	1	7	1	0	1	7	7,1	139,88	1952800	0	2262000	5728500	0	44,706
Q6PFR5	Q6PFR5	Transformer-2 protein homolog alpha	Tra2a	0	0	1	1	0	0	1	1	5	32,316	0	0	5985700	886240	0,00098232	7,3243
P62996	P62996	Transformer-2 protein homolog beta	Tra2b	0	0	1	2	0	0	1	2	10,1	33,665	0	0	268310	710940	0	13,428
Q62318	Q62318	Transcription intermediary factor 1-beta	Trim28	3	1	2	5	3	1	2	5	10	88,846	1512000	144060	5635500	1,33E+07	0	40,426
Q3TX08	Q3TX08	tRNA (guanine(26)-N(2))-dimethyltransferase	Trmt1	1	0	1	2	1	0	1	2	3,3	72,349	1028000	0	2584700	1540400	0	12,077
Q5SWD9	Q5SWD9	Pre-rRNA-processing protein TSR1 homolog	Tsr1	1	0	2	5	1	0	2	5	8,7	92,104	91824	0	8160100	6614600	0	139,12
P23591	P23591	GDP-L-fucose synthase	Tsta3	1	0	1	1	1	0	1	1	8,7	35,877	1565600	0	1243600	531020	0	55,233
P68372;Q9D6F9	P68372;Q9D6F9	Tubulin beta-4B chain;Tubulin beta-4A chain	Tubb4b;Tubb4a	14	6	18	18	2	1	2	4	49,9	49,83	8,94E+07	3154100	4,52E+08	1,25E+08	0	323,31
P99024;Q9CWF2;Q7TMM9	P99024;Q9CWF2;Q7TMM9	Tubulin beta-5 chain;Tubulin beta-2B chain;Tubulin beta-2A chain	Tubb5;Tubb2b;Tubb2a	14	7	18	16	2	1	2	2	45,7	49,67	3,37E+07	1685100	1,64E+08	4,41E+07	0	47,368
ENSEMBL:ENSBTAP00000(	Q922F4	Tubulin beta-6 chain	Tubb6	8	3	12	10	3	2	3	2	33,3	50,09	4680300	427290	6,19E+07	9506800	0	116,75
Q6NV83	Q6NV83	U2 snRNP-associated SURP motif-containing protein	U2surp	0	0	1	1	0	0	1	1	2,1	118,26	0	0	2478600	318610	0	13,753
Q3TW96;Q91YN5;Q6P549	Q3TW96	UDP-N-acetylhexosamine pyrophosphorylase-like protein 1	Uap1l1	6	3	6	3	6	3	6	3	15,6	56,613	1,42E+07	1099100	3,69E+07	7229000	0	52,679
Q02053;P31254	Q02053	Ubiquitin-like modifier-activating enzyme 1	Uba1	8	4	6	16	8	4	6	16	23,2	117,81	2,40E+07	694330	8,58E+07	7,50E+07	0	323,31
Q80X50	Q80X50	Ubiquitin-associated protein 2-like	Ubap2l	1	0	1	4	1	0	1	4	5,7	116,8	4517000	0	911060	2,28E+07	0	91,148
P61082	P61082	NEDD8-conjugating enzyme Ubc12	Ube2m	1	0	1	2	1	0	1	2	10,9	20,9	528830	0	2743700	706560	0	11,577
Q9ES00	Q9ES00	Ubiquitin conjugation factor E4 B	Ube4b	1	0	1	1	1	0	1	1	2,3	133,32	225990	0	431500	649100	0	12,695
A2AN08	A2AN08	E3 ubiquitin-protein ligase UBR4	Ubr4	0	0	2	9	0	0	2	9	2,4	572,28	0	0	2677200	7011300	0	62,311
P13439	P13439	Uridine 5-monophosphate synthase;Orotate phosphoribosyltransferase;Orotidine 5-phosphate decarboxylase	Umps	0	0	2	2	0	0	2	2	6,7	52,292	0	0	4781600	1036200	0	22,034
Q9EPU0	Q9EPU0	Regulator of nonsense transcripts 1	Upf1	0	0	4	6	0	0	4	6	8,3	123,97	0	0	1,28E+07	2,21E+07	0	48,556
Q9Z1Z0	Q9Z1Z0	General vesicular transport factor p115	Uso1	1	0	2	3	1	0	2	3	4,4	106,98	45464	0	1280500	2260400	0	48,201

Q9JMA1	Q9JMA1	Ubiquitin carboxyl-terminal hydrolase 14	Usp14	1	0	1	3	1	0	1	3	9,7	56,001	671960	0	2311800	2806500	0	20,786
Q8C7V3	Q8C7V3	U3 small nucleolar RNA-associated protein 15 homolog	Utp15	0	0	1	2	0	0	1	2	6,8	59,374	0	0	2230100	1974800	0	48,291
Q5XG71	Q5XG71	Small subunit processome component 20 homolog	Utp20	0	0	1	5	0	0	1	5	2,2	317,74	0	0	1150600	2466200	0	44,098
Q80WQ2	Q80WQ2	Protein VAC14 homolog	Vac14	0	0	1	2	0	0	1	2	4,7	88,047	0	0	922060	894460	0	24,773
P63024;P63044	P63024;P63044	Vesicle-associated membrane protein 3;Vesicle-associated membrane protein 2	Vamp3;Vamp2	1	1	1	2	1	1	1	2	32	11,48	204520	44984	212000	579490	0	13,152
Q9Z1Q9	Q9Z1Q9	Valine--tRNA ligase	Vars	6	0	15	25	6	0	15	25	26,4	140,21	1,12E+07	0	4,19E+07	1,30E+08	0	323,31
P70460	P70460	Vasodilator-stimulated phosphoprotein	Vasp	2	0	3	2	2	0	3	2	16,8	39,666	894410	0	6794400	6182100	0	28,695
Q62465	Q62465	Synaptic vesicle membrane protein VAT-1 homolog	Vat1	3	0	2	7	3	0	2	7	20,7	43,096	4741700	0	7263800	4,66E+07	0	162,34
Q91W86	Q91W86	Vacuolar protein sorting-associated protein 11 homolog	Vps11	0	0	1	1	0	0	1	1	1,6	107,72	0	0	1713000	467820	0,00096525	7,2429
Q8BX70	Q8BX70	Vacuolar protein sorting-associated protein 13C	Vps13c	1	0	1	13	1	0	1	13	4,5	420,08	77179	0	756040	1,21E+07	0	96,672
P40336	P40336	Vacuolar protein sorting-associated protein 26A	Vps26a	1	0	1	4	1	0	1	4	18,3	38,113	758680	0	2329100	3936600	0	42,442
Q9QZ88	Q9QZ88	Vacuolar protein sorting-associated protein 29	Vps29	1	0	1	1	1	0	1	1	7,1	20,495	239860	0	453230	252110	0,00090909	6,655
Q9EQH3	Q9EQH3	Vacuolar protein sorting-associated protein 35	Vps35	4	1	5	11	4	1	5	11	17,2	91,712	1,43E+07	172980	5,98E+07	2,17E+07	0	293,22
Q3UVL4	Q3UVL4	Vacuolar protein sorting-associated protein 51 homolog	Vps51	0	0	2	1	0	0	2	1	5	86,186	0	0	1974700	392800	0	21,967
Q80X41	Q80X41	Serine/threonine-protein kinase VRK1	Vrk1	1	0	1	2	1	0	1	2	6,8	49,74	124790	0	3666500	2101400	0	12,929
Q99KC8	Q99KC8	von Willebrand factor A domain-containing protein 5A	Vwa5a	2	0	2	5	2	0	2	5	8,6	87,142	1284600	0	2297900	4369500	0	40,333
P32921	P32921	Tryptophan--tRNA ligase, cytoplasmic;T1-TrpRS;T2-TrpRS	Wars	0	0	2	12	0	0	2	12	36,8	54,357	0	0	1391800	2,62E+07	0	111,38
Q6ZQL4	Q6ZQL4	WD repeat-containing protein 43	Wdr43	1	0	2	3	1	0	2	3	9	75,38	133820	0	6388400	2620600	0	48,435
Q6P5F9	Q6P5F9	Exportin-1	Xpo1	1	0	1	9	1	0	1	9	10,4	123,09	188080	0	1760400	9137300	0	90,849
Q924C1	Q924C1	Exportin-5	Xpo5	0	0	1	3	0	0	1	3	3	136,97	0	0	2765300	1704200	0	19,751
Q9CRT8	Q9CRT8	Exportin-T	Xpot	0	0	1	2	0	0	1	2	2,8	109,73	0	0	2090800	2041600	0	13,362
P62960	P62960	Nuclease-sensitive element-binding protein 1	Ybx1	2	0	1	3	2	0	1	3	24,5	35,73	741080	0	8934300	2472200	0	27,254
P68254	P68254	14-3-3 protein theta	Ywhaq	0	0	1	1	0	0	1	1	5,7	27,778	0	0	457870	90867	0,0017528	6,4652
P63101	P63101	14-3-3 protein zeta/delta	Ywhaz	0	0	2	1	0	0	2	1	9,4	27,771	0	0	4240700	58980	0	64,447
Q3UPF5	Q3UPF5	Zinc finger CCCH-type antiviral protein 1	Zc3hav1	1	0	1	1	1	0	1	1	1,5	106,69	344160	0	2079400	522710	0	47,219
Q8BWQ6	Q8BWQ6	UPF0505 protein C16orf62 homolog		1	0	1	1	1	0	1	1	2,4	109,08	263400	0	2410000	1224200	0	11,791

[a] Herein, and for all other columns, we followed the definitions and parameters as in MaxQuant (ver. 1.6.0.1). Identifier(s) of protein(s) contained in the Protein Group created by the built-in Andromeda searching

[b] When the same peptide sequences is assigned to diverse proteins in the reference database, here only the ID of proteins with a more confident identification is reported for sake of clarity

[c] Total number of peptide sequences associated with all proteins in the group in treated (Aβ1-Aβ2) and untreated (NT1-NT2) EVs samples.

[d] Total number of peptide sequences exclusively assigned to the protein group in treated (Aβ1-Aβ2) and untreated (NT1-NT2) EVs samples. In this study, we accepted from the original data set only identification achieved with at least 1

[e] Sequence coverage of the best protein sequence contained in the group by the identified peptides, expressed as %.

[f] Molecular weight of the leading protein sequence contained in the protein group.

[g] Summed up the eXtracted Ion Current of all isotopic clusters associated con with the identified aminioacid sequences for each protein group in in treated (Aβ1-Aβ2) and untreated (NT1-NT2) EVs samples.

[h] In the MaxQuant algorithms, Q-values of a protein group are the name given to the adjusted p-values found using an optimised FDR approach. It ranges between 0 and 1, with higher values referring for an high probability to be an fault positive hit.

[i] Score for the protein identification, determined by multiplying each peptide posterior error probabilities (PEPs) calculated by the Andromeda search engine for assigning statistical significance to the peptide identifications. The larger this score, the more certain is the identification of a protein.
