# Supplementary material for: Rab11A Depletion in Microglia-Derived Extracellular Vesicle Proteome upon Beta-Amyloid Treatment
Source: Cell Biochem Biophys. 2023 Mar 30;81(2):337–47. doi: 10.1007/s12013-023-01133-4 (PMC10257621; doi:10.1007/s12013-023-01133-4)
Supplement: Supplementary file 3 — Supplementary Information [file 12013_2023_1133_MOESM3_ESM.docx]

**Fig. 1S** 4-12% SDS-PAGE of EV samples untreated (NT1and NT2) and treated (Aβ1 and Aβ2) with 25µM Aβ_25–35_ for 24 h. Loaded aliquots were chosen to rely on the protein content of cell lysates where a similar amount of protein was detected. Lanes marked with * are biological replicates not used in this study for technical problems
